# Targeting PEG10 as a novel therapeutic approach to overcome CDK4/6 inhibitor resistance in breast cancer

**DOI:** 10.1186/s13046-023-02903-x

**Published:** 2023-11-28

**Authors:** Nar Bahadur Katuwal, Min Sil Kang, Mithun Ghosh, Sa Deok Hong, Yeong Gyu Jeong, Seong Min Park, Seul-Gi Kim, Joohyuk Sohn, Tae Hoen Kim, Yong Wha Moon

**Affiliations:** 1 Department of Biomedical Science, The Graduate School, CHA University, Seongnam-Si, 13488 Republic of Korea; 2 Hematology and Oncology, Department of Internal Medicine, CHA Bundang Medical Center, CHA University, 59 Yatap-Ro, Bundang-Gu, Seongnam-Si, Gyeonggi-Do 13496 Republic of Korea; 3 Division of Medical Oncology, Department of Internal Medicine, Yonsei Cancer Center, Yonsei, University College of Medicine, Seoul, 03080 Korea; 4 Department of Pathology, CHA Bundang Medical Center, CHA University, Seongnam-Si, 13496 Republic of Korea

**Keywords:** CDK4/6, ASO, PEG10, HR+ breast cancer, Drug resistance

## Abstract

**Background:**

Breast cancer is the global leading cancer burden in women and the hormone receptor-positive (HR+) subtype is a major part of breast cancer. Though cyclin-dependent kinase 4 and 6 (CDK4/6) inhibitors are highly effective therapy for HR+ subtype, acquired resistance is inevitable in most cases. Herein, we investigated the paternally expressed gene 10 (PEG10)-associated mechanism of acquired resistance to CDK4/6 inhibitors.

**Methods:**

Palbociclib-resistant cells were generated by exposing human HR+ breast cancer cell lines to palbociclib for 7–9 months. In vitro mechanistic study and in vivo xenograft assay were performed. For clinical relevance, public mRNA microarray data sets of early breast cancer were analyzed and PEG10 immunohistochemical staining was performed using pre-CDK4/6 inhibitor tumor samples.

**Results:**

We observed that PEG10 was significantly upregulated in palbociclib-resistant cells. Ectopic overexpression of PEG10 in parental cells caused CDK4/6 inhibitor resistance and enhanced epithelial–mesenchymal transition (EMT). On the contrary, PEG10-targeting siRNA or antisense oligonucleotides (ASOs) combined with palbociclib synergistically inhibited proliferation of palbociclib-resistant cells and growth of palbociclib-resistant xenograft in mice and suppressed EMT as well. The mechanistic study confirmed that high PEG10 expression suppressed p21, a natural CDK inhibitor, and SIAH1, a post-translational degrader of ZEB1, augmenting CDK4/6 inhibitor resistance. Then PEG10 siRNA combined with palbociclib suppressed cell cycle progression and EMT via activating p21 and SIAH1, respectively. Consequently, combined PEG10 inhibition and palbociclib overcame CDK4/6 inhibitor resistance. Furthermore, high PEG10 expression was significantly associated with a shorter recurrence-free survival (RFS) based on public mRNA expression data. In pre-CDK4/6 inhibitor treatment tissues, PEG10 positivity by IHC also showed a trend toward a shorter progression-free survival (PFS) with CDK4/6 inhibitor. These results support clinical relevance of PEG10 as a therapeutic target.

**Conclusions:**

We demonstrated a novel PEG10-associated mechanism of CDK4/6 inhibitor resistance. We propose PEG10 as a promising therapeutic target for overcoming PEG10-associated resistance to CDK4/6 inhibitors.

**Supplementary Information:**

The online version contains supplementary material available at 10.1186/s13046-023-02903-x.

## Background

Breast cancer is the leading cause of cancer burden with 2.3 million new cases and 685,000 deaths from cancer among women globally in 2020 [1]. Among the three breast cancer subtypes, hormone receptor-positive (HR+), human epidermal growth factor receptor 2-positive (HER2+), and triple-negative subtypes, the HR+ subtype comprises about 70% of all breast cancers [2]. Although endocrine therapy is an effective treatment mostly for the HR+ breast cancer, acquired resistance becomes a critical issue in many cases [3].

Dysregulation of cyclin-dependent kinases (CDKs), mainly CDK4/6 and retinoblastoma protein (RB) pathway induces sustained cellular proliferation and has been associated with the pathogenesis of HR+ breast cancer [4]. The RB tumor suppressor protein directly binds to and blocks the E2F transactivation domain and represses E2F transcriptional activity. However, phosphorylation of RB by the cyclin D-CDK4/6 complex impairs the interaction with E2F, promoting cell cycle progression and contributing to cancer development [5]. Therefore, CDK4/6 inhibitors combined with endocrine therapy have emerged as a main treatment strategy for HR+/HER2– breast cancer as a first or subsequent line of therapy.

Despite the excellent outcome of CDK4/6 inhibitor therapy, acquired resistance emerges in almost all cases after 24–28 months post-first-line therapy [6] and after a shorter duration post-second-line therapy [7, 8]. Several studies demonstrated that loss of RB [4] or amplification of CDK6 [9], CDK4 [10], cyclin E [11], and p16 [12] may cause resistance to CDK4/6 inhibitors. In addition, activation of bypass pathways such as FGFR [13] or PI3K/AKT/mTOR [14] is reported to be a potential mechanism of CDK4/6 inhibitor resistance. Accordingly, research has focused on overcoming CDK4/6 inhibitor resistance by targeting these pathways [15, 16]. However, strategies to overcome resistance have not yet been established. Therefore, studies on novel targets should be pursued.

Based on our previous results that expression of epithelial–mesenchymal transition (EMT)-associated genes were upregulated in palbociclib-resistant cells [6], we searched for EMT-regulating genes that may be associated with CDK4/6 inhibitor resistance. Paternally expressed gene 10 (PEG10) has gained attention as this has been reported to augment the EMT [17] and can mediate cell proliferation, invasion, metastasis, and potential drug resistance [18–20]. Additionally, upregulated expression of PEG10 has been observed in malignancies such as hepatocellular carcinoma, bladder cancer, lung cancer, breast cancer, pancreatic cancer [21], and cutaneous T-cell lymphoma [20]. PEG10 is a human retrotransposon-derived imprinted gene located at human chromosome 7q21 [22] and plays a crucial role in placenta formation [23], and adipocyte differentiation [24]. In addition, the knockout of this gene causes embryonic lethality [23]. Upregulated PEG10 expression is involved in oncogenesis and cancer progression as mentioned above, prompting us to investigate whether PEG10 was involved in CDK4/6 inhibitor resistance.

Based on eventual development of CDK4/6 inhibitor resistance and there being no approved therapies to overcome the resistance, we established a preclinical model with palbociclib-resistant breast cancer to reveal mechanisms of CDK4/6 inhibitor resistance. A subsequent screening assay discovered PEG10 as a top candidate gene. Therefore, in this study, we investigated PEG10-associated mechanism of CDK4/6 inhibitor resistance. Furthermore, we provide a scientific rationale of PEG10 as a promising therapeutic target for overcoming PEG10-associated resistance to CDK4/6 inhibitor.

## Methods

### Drugs

Palbociclib (cat# CS-0019), abemaciclib (cat# CS-1230) and ribociclib (cat# CS-1750) were purchased from ChemScene (Monmouth Junction, NJ, USA). Palbociclib was dissolved in water while all other drugs were reconstituted in dimethyl sulfoxide (DMSO) (Sigma-Aldrich, USA).

### Cell culture

HR+ breast cancer cell lines MCF7, T47D, and endogenously PEG10-expressing prostate cancer cell line PC3 were purchased from the American Type Culture Collection (Manassas, VA, USA) and maintained in a humidified atmosphere with 5% CO_2_ at 37 °C. Parental (MCF7 and T47D) and palbociclib-resistant cells (MCF7-PR and T47D-PR) were cultured in RPMI 1640 medium (cat# LM011-01, Welgene Inc., Daegu, Korea) supplemented with 10% heat-inactivated FBS (cat# S 001–01; Welgene Inc., Daegu, Korea) and 1% 100 × P/S solution (cat# LS202-02, Welgene Inc. Daegu, Korea). All the palbociclib-resistant cells were maintained with 5 µM palbociclib, and fresh medium was changed before 24–48 h of experiments.

### Generation of resistant cells

Palbociclib-resistant cells (MCF7-PR and T47D-PR) were established after 7 − 9 months by a gradual increase in the palbociclib concentration starting from the IC_50_, which was 750 and 250 nM for MCF7 and T47D cells, respectively. Detailed methods for generation of palbociclib-resistant cells are explained in our previous study [6].

### Gene expression microarray analysis

Gene expression microarray analysis was performed as described previously [6]. In brief, to discover the differentially expressed genes (DEGs), we performed microarray of palbociclib-resistant (MCF7-PR and T47D-PR) versus their corresponding parental (MCF7 and T47D) cells. First, RNA samples were extracted. Then Affymetrix Whole Transcript Expression array process was performed according to the manufacturer’s protocol (GeneChip Whole Transcript PLUS reagent Kit, ThermoFisher Scientific, USA). Signal values were computed using the Affymetrix® GeneChip™ Command Console software. Genes exhibiting at least a two-fold upregulation or downregulation were considered meaningful.

### Quantitative real-time polymerase chain reaction (qRT-PCR)

qRT-PCR was performed on total RNA isolated from palbociclib-resistant (MCF7-PR and T47D-PR) and their corresponding parental (MCF7 and T47D) cells to validate the mRNA expression level. cDNA was synthesized using the Takara prime script 1^st^ strand cDNA synthesis kit (cat# 6110A, Takara Bio Inc., Japan) according to the manufacturer’s instructions. qRT-PCR was performed using a ViiA7 Real-time PCR system (Applied Biosystems, Warrington, UK) and a Power-up SYBR Green Master Mix (cat# A25741, ThermoFisher Scientific, USA). The target gene expression was normalized to the β-actin. A list of PCR primers is listed in Supplementary Table S1.

### Western blots

Cell protein was extracted with RIPA Lysis Buffer (cat# 89901, ThermoFisher Scientific, USA) combined with a protease inhibitor cocktail (cat# 11873580001, Roche, Germany) and phosphatase inhibitors (cat# 1862495, ThermoFisher Scientific, USA), and the concentration was detected by Pierce™ BCA protein assay (cat# 23228 and cat# 1859078, ThermoFisher Scientific, USA) Kit. Western blotting was performed as described previously [6]. The antibodies used for western blotting are listed in Supplementary Table S2.

### Genomics of Drug Sensitivity in Cancer (GDSC) database analysis

Gene expression and drug screening data for the cell lines were downloaded from the GDSC website [25]. A total of 43 breast cancer cell lines were used to analyze the association between PEG10 expression and palbociclib sensitivity. Furthermore, among the 43 breast cancer cell lines, a subset comprising 11 HR+ breast cancer cell lines were also used to analyze the association between PEG10 expression and palbociclib sensitivity. Palbociclib sensitivity was defined as IC_50_ of < 3.5 µM. The list of 43 breast cancer cell lines is shown in the Supplementary Table S3.

### Plasmids and ectopic overexpression studies

PEG10 has two overlapping open reading frames (ORFs), where the translation of ORF1 produces the PEG10 (RF1) gag-like protein that is associated with invasion and drug resistance, whereas the pol-like PEG10 (RF2) protein is synthesized by a programmed –1 frameshift translation [21, 26].

PEG10 plasmid, (PEG10-RF1 and PEG10-fsRF1/RF2), was generously provided by Prof. Dr. Jozsef Tozser, University of Debrecen, Hungary. Flag p21 WT plasmid (Plasmid # 16240) [27] was purchased from Addgene, and pCMV-SPORT6/SIAH1 plasmid (clone number hMU004814) was purchased from Korea Human Gene Bank (Medical Genomics Research Center, KRIBB, Korea), and respective empty vectors were used for MOCK controls. 5 × 10^5^ cells were seeded, and the next day plasmid was transfected using the CalFectin ™ Mammalian DNA Transfection Reagent (cat# SL1000478, SignaGen Laboratories, USA) following the manufacturer’s instructions. After 48 h, cells were collected, and overexpression was confirmed using western blotting.

### Migration and invasion assay

Migration and invasion assay was performed as described previously [28]. In brief, for migration and invasion assay, 5 × 10^5^ cells were seeded in insert well with serum-free medium and incubated for 24–48 h at 37 °C. For the invasion assay, 50–100 µL of Matrigel matrix (cat# 254234, Corning, NY, USA) was coated in the insert well before cell seeding.

After incubation for the indicated time, the media was aspirated and unmigrated and non-invaded cells were carefully removed using cotton from the insert well. Furthermore, migrated, and invaded cells were fixed with 2% PFA, and crystal violet staining was performed for 5–10 min. Washed with PBS and water and then dried for 3–4 h before being observed under a light microscope. ImageJ software version 1.53t was used to quantify the area of migratory and invasive cells in five random nonoverlapping microscopic fields of each insert well at 100 × magnification.

### Cell viability assay

Cell viability was measured using the thiazolyl blue tetrazolium bromide (MTT; Sigma, St. Louis, MO, USA) assay as previously described [6]. A total of 1,000–1,500 cells per well were seeded in 96-well plates and incubated overnight. Cells were then treated with various concentrations of palbociclib and fixed concentration of PEG10 siRNA (cat# 4392421, Assay ID: s23005, ThermoFisher Scientific, USA) with Lipofectamine RNAiMAX (cat# 13778100, ThermoFisher Scientific, USA) or PEG10-antisense oligonucleotide (PEG10-ASO) with Lipofectamine 2000 (cat# 11668019, ThermoFisher Scientific, USA) in OptiMEM® I reduced-serum medium (cat# 31985070, ThermoFisher Scientific, USA) and incubated for 72 h at 37 °C. Subsequent procedures were performed as described in our previous study [6]. The IC_50_ and combination index (CI) values were analyzed using CompuSyn software (ComboSyn, NJ, USA).

### Double thymidine block cell cycle and fluorescence-activated cell sorting analysis (FACS)

For the double thymidine block experiment, cells were seeded at 5 × 10^5^ in a 60-mm cell culture dish. After overnight incubation, 2 mM thymidine (cat# sc-296542, Santa Cruz Biotechnology, USA) was added for 18 h. Media was replaced with fresh complete media for 9 h; a second thymidine block was then performed for 18 h. Finally, cells were treated with 25 nmol PEG10 siRNA in a time-dependent manner, and cell cycle analysis was performed as described previously [6]. In brief, harvested cells were fixed with 70% ethanol, resuspended in RNAse A (100 μg/ml), and stained with propidium iodide (50 μg/ml) in PBS. Stained cells were assessed using a CytoFLEX flow cytometer (Beckman Coulter, USA), data were analyzed using FlowJo v10 (FlowJo LLC, Ashland, OR 97520, USA), and the percentage of cells in each phase of the cell cycle was determined.

### Gene knockdown experiments

To perform siRNA/shRNA knockdown experiments, cells were seeded at 5 × 10^5^ in a 60-mm cell culture dish and allowed to attach overnight. After overnight incubation, cells were transfected with scramble siRNA (cat# Sc-37007, Santa Cruz Biotechnology, USA) or PEG10 siRNA using Lipofectamine RNAiMAX reagent and ZEB1 shRNA (TRC Number: TRCN0000369267, Sigma-Adrich INC) by using Lipofectamine 2000 reagent following the manufacturer’s instructions. After 24–48 h of incubation, cells were isolated and analyzed for further experiments.

To perform ASO knockdown experiments, 5 different PEG10-ASOs and control-ASO were designed and synthesized by Creative Biolabs (USA). PEG10-ASOs were designed with a central gap segment comprising ten 2ʹ-deoxynucleosides and a central gap that was flanked on the 5ʹ and 3ʹ wings by five 2ʹ-MOE modified nucleosides; all internucleoside linkages were phosphorothioate linkages. For in vitro screening of PEG10-ASOs to select the most effective ASO, 5 × 10^5^ cells were seeded in a 60-mm cell culture dish and allowed to attach overnight. Various doses of each ASO (100–500 nmol) were then diluted in 200 µL of OptiMEM® I reduced-serum medium and Lipofectamine 2000 reagent was diluted separately in 200 µL of OptiMEM® I reduced-serum medium, according to the manufacturer’s instructions. Diluted ASOs and Lipofectamine were mixed and incubated for 15 min at room temperature and added to the cells dropwise and then incubated for various times from 24–72 h. Cells were harvested for western blotting and qRT-PCR analysis. The best candidate ASO among the 5 different PEG10-ASOs was selected for further experiments such as in vivo efficacy assay according to the PEG10 knockdown efficacy. The sequence of control-ASO used was (5ʹ CCUUC CCTGAAGGTT CCUCC 3ʹ), which was adopted from Worley et al. [29]. The sequence of the best candidate PEG10-ASO was (5ʹ UUUGG TGCTTTAGGA UGUGU 3ʹ).

### Animal studies

For tumor xenograft experiments, 5-week-old female BALB/c-nude mice were purchased from Gem Biosciences (Cheongju, Korea) and kept in a pathogen-free environment. All animal procedures were performed following the IACUC-approved protocol of CHA University. A total of 1 × 10^7^ MCF7-PR cells in 0.1 mL of PBS containing a 50% (v/v) Matrigel solution (cat# 354248, Corning, NY, USA) were subcutaneously injected into the mammary fat pad of the mouse. For the estrogen supplement, estrogen valerate (3 μg/mouse) was subcutaneously injected once a week. When the tumor volume reached 60–80 mm^3^, the animals were randomized into four groups, each containing five mice. After grouping, mice were treated with control-ASO 15 mg/kg (group 1), palbociclib 100 mg/kg (group 2), PEG10-ASO 15 mg/kg (group 3), and PEG10-ASO with palbociclib (group 4). ASO was dissolved in PBS and injected systematically via intraperitoneal injection once per day for 5 days and then three times per week there after for a total of 18 days. Palbociclib was administered by oral gavage once daily for 22 days. Tumor size was measured three times a week using a vernier caliper, and the volume was calculated by using the formula (tumor length × tumor width^2^ × 0.5). Additionally, body weight was regularly measured three times a week. At 22 days, all mice were euthanized and the xenografted tumors were excised and preserved for further analysis.

### Immunohistochemistry (IHC) staining analysis

We performed the IHC using the xenograft tissues to assess the PEG10 and Ki67 expression, and also using the patients’ tumor samples to assess PEG10 expression. Firstly, to perform the IHC using the xenograft tissues, samples were deparaffinized and antigen retrieval was performed using heat-mediated methods with an IHC antigen retrieval solution (cat# 00–4955-58, Invitrogen, ThermoFisher Scientific, USA) for 25 min. The section was stained with PEG10 primary antibody (cat# 14412–1-AP, Proteintech, USA) or Ki67 primary antibody (cat# ab92742, abcam, USA) at 1:200 dilution for 12 h at 4 °C. Next day, the section was washed, and HRP-conjugated secondary antibody was added for 1 h at room temperature. Finally, slides were counterstained, and mounting was performed, and then examined under a light microscope. To quantify IHC staining, the number of positively stained cells were counted using ImageJ software version 1.53t in five random nonoverlapping 400 × microscopic fields for each tissue section.

Secondly, to evaluate if the PEG10 protein expression level is associated with efficacy of CDK4/6 inhibitor, we performed the IHC using the patients’ tumor samples. Patients’ formalin-fixed, paraffin-embedded tumor samples were prepared from 15 patients with advanced breast cancer. Clinical characteristics of these patients were listed in the Supplementary Table S4. Fourteen patients were HR+/HER2– and one HR+/HER2+. Regarding metastatic sites in the beginning of CDK4/6 inhibitor treatment, 10 patients (66.7%) had visceral metastases. All these patients were treated with combined palbociclib and letrozole or anastrozole as a first-line therapy. During median follow-up of 10 months (1.0–72.5), 11 patients stopped CDK4/6 inhibitor treatment due to disease progression (*n* = 10) and adverse events (*n* = 1). All tumor specimens were obtained before CDK4/6 inhibitor treatment at CHA Bundang Medical Center (Seongnam, South Korea). For IHC of patients’ tumor samples, the automatic device Ventana Discovery ULTRA Stainer (Roche, Germany) was used, and the procedure was performed according to the manufacturer’s instructions. In brief, human tissue sections were stained with PEG10 primary antibody at 1:200 dilution (cat# 14412–1-AP, Proteintech, USA) for 60 min. Following incubation, one drop OmniMap anti-Rb HRP (cat# 760–4310, Roche, USA) was added and incubated for 16 min. Finally, after counterstain mounting was performed. Positivity for PEG10 was assessed from 0 to 100% of stained cells by cytoplasmic staining with any intensity. The cut-off of 1% was used for PEG10 positivity as in a previous report [30]. CDK4/6 inhibitor progression-free survival (PFS) was analyzed according to PEG10 positivity.

### Terminal deoxynucleotidyl transferase dUTP nick end labeling (TUNEL) assay

TUNEL assay was conducted to evaluate apoptosis in xenograft tissues according to the manufacturer’s instructions (cat# S7101, Merck, MA, USA). Briefly, after dewaxing and hydration, slides were treated with TUNEL and hematoxylin solution for indicated times. To quantify TUNEL assay results, the number of positively stained cells were counted using ImageJ software version 1.53t in five random nonoverlapping 400 × microscopic fields for each tissue section.

### Public gene expression profiling data sets in patients with breast cancer

To evaluate if the PEG10 mRNA expression level is associated with prognosis in patients with HR+ breast cancer, we used public mRNA expression data sets of curatively resected HR+ breast cancer. Firstly, of downloadable public data sets, we searched for data sets of minimum 200 estrogen receptor–positive (ER+) patients containing essential clinical factors such as pathologic stages and recurrence to conduct multivariate analyses. We chose GSE 6532 and series matrix files of the data set, which were already normalized by original authors [31], were downloaded for our analyses. Secondly, other data sets were analyzed using online platform Kaplan–Meier plotter [32] (https://kmplot.com/analysis/) without downloading processed raw data. Of the total 24 data sets analyzed, we selected 4 data sets (GSE 2034, E-MTAB-365, GSE 3494, and GSE 25066) with the criteria of ER+ status by IHC and minimum 200 analyzed patients. Affymetrix GeneChip Human Genome U133 Arrays were used for all these 5 GSE data sets. Affymetrix ID 212092 was selected for PEG10. PEG10 mRNA expression level was divided into two groups, “PEG10-High” and “PEG10-Low” with cut-off of upper quartile (GSE 6532) or auto-select best cut-off (GSE 2034, E-MTAB-365, GSE 3494, and GSE 25066). An optimal cut-off point for normalized intensity of PEG10 mRNA was determined using minimum *p*-value approach in predicting recurrence-free survival (RFS) [33].

### Statistical analysis

All statistical analysis were performed using SPSS version 19.0 (IBM SPSS statistics 19.0, NY, USA). Student’s *t*-test was used to analyze independent two groups comprising continuous variables: the qRT-PCR of mRNA expression of EMT genes, gene expression according to palbociclib sensitivity using GDSC database, cell viability assay, cell cycle analysis, and tumor growth inhibition. IHC, TUNEL assay, invasion, and migration assay data were first quantified using ImageJ software version 1.53t, and *p*-values were analyzed by Student’s *t*-test using quantified data. Values are represented as mean ± SD unless otherwise indicated. RFS was defined as the time from curative surgery to breast cancer recurrence or the last date when the patient was known to be free of recurrence (censoring time). PFS was defined as the time from CDK4/6 inhibitor treatment to breast cancer progression or the last date when the patient was known to be free of progression (censoring time). RFS and PFS were calculated using the Kaplan–Meier method. A log-rank test was used to compare RFS and PFS between groups. Significant prognostic variables by univariate analysis, defined as those with a *p*-value < 0.05 were submitted to multivariate analysis using the Cox proportional hazard regression model. All *p-*values were two-tailed, and *p-*values < 0.05 were considered as significant.

## Results

### Characterization of palbociclib-resistant cells with high PEG10 expression and EMT process activation

**Fig. 1:**
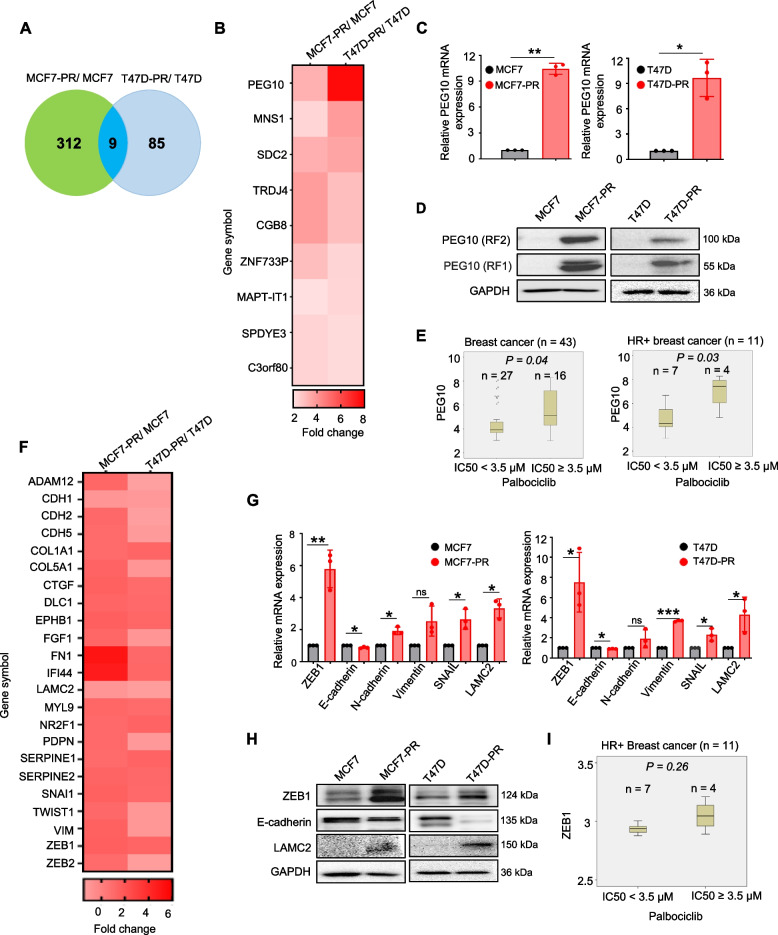
Characterization of palbociclib-resistant cells with high PEG10 expression and EMT process activation. **A** Venn diagram representing the number of upregulated genes in MCF7-PR and T47D-PR cells. A gene showing fold change ≥ 2 compared with that in parental cells (MCF7 and T47D) is considered an upregulated gene. **B** Heat map showing the list of commonly upregulated genes in MCF7-PR and T47D-PR cells. **C** mRNA expression of PEG10 in the palbociclib-resistant (MCF7-PR and T47D-PR) compared with their corresponding parental (MCF7 and T47D) cells by qRT-PCR. Three independently repeated experiments were performed with similar results. Independent sample t-test: *p < 0.05, **p < 0.01. **D** Immunoblots showing PEG10 (RF1) and PEG10 (FR2) protein expression in the palbociclib-resistant (MCF7-PR and T47D-PR) and parental (MCF7 and T47D) cells. **E** Association of PEG10 expression and palbociclib sensitivity using GDSC database. Palbociclib sensitivity was defined as IC_50_ ≤ 3.5 µM. *P-*value was calculated by independent sample t-test. **F** A panel of genes associated with EMT process from microarray data analysis in palbociclib-resistant (MCF7-PR and T47D-PR) versus parental (MCF7 and T47D) cells. **G** mRNA expression of EMT markers in the palbociclib-resistant (MCF7-PR and T47D-PR) cells compared with their corresponding parental (MCF7 and T47D) cells by qRT-PCR. Three independently repeated experiments were performed with similar results. Independent sample t-test: *p < 0.05, **p < 0.01, ***p < 0.001, Abbreviation: ns, not significant. **H** Immunoblots showing protein expression of mesenchymal markers (ZEB1 & LAMC2), and epithelial marker (E-cadherin) in MCF7-PR and T47D-PR cells compared with MCF7 and T47D cells, respectively. **I** Association of ZEB1 expression and palbociclib sensitivity in 11 HR+ breast cancer cell lines from GDSC database. Palbociclib sensitivity was defined as IC_50_ ≤ 3.5 µM. *P-*value was calculated by independent sample t-test

Palbociclib-resistant cells were generated by culturing MCF7 and T47D cells with increasing concentrations of palbociclib. We previously demonstrated that palbociclib-resistant cells were cross-resistant to other CDK4/6 inhibitors (abemaciclib and ribociclib) as confirmed with cell viability (MTT) and cell cycle assays [6]. To identify DEGs in palbociclib-resistant cells compared with their corresponding parental cells, microarray analysis was performed using palbociclib-resistant and their corresponding parental cells. A total of 31,135 genes were analyzed by microarray. Of those genes, we selected ones that exhibited more than two-fold change in expression level compared with their corresponding parental cells, which were defined as DEGs. Notably among these DEGs, 9 genes were shared in both palbociclib-resistant cells (MCF7-PR, T47D-PR); that is, PEG10, MNS1, SDC2, TRDJ4, CGB8, ZNF733P, MAPT-IT1, SPDYE3 and C3orF80 were commonly upregulated in both palbociclib-resistant cells compared with their corresponding parental cells (Fig. 1A). PEG10 was the top gene among the 9 shared DEGs in both palbociclib-resistant cells (Fig. 1B); detailed fold changes of those 9 genes are provided in the Supplementary Table S5. Upregulation of PEG10 gene and protein levels in palbociclib-resistant cells was confirmed with qRT-PCR and western blotting (Fig. 1C and D), respectively. Moreover, the association between high PEG10 expression and palbociclib resistance was supported by analysis of the GDSC database. From data for 43 breast cancer cell lines, we determined that cells with low palbociclib activity (at half the maximal inhibitory concentration [IC_50_] > 3.5 µM) had higher PEG10 expression (*p* = 0.04) (Fig. 1E left panel). A similar association was seen in the smaller subset of 11 HR+ cell lines (*p* = 0.03) (Fig. 1E right panel). These results suggest that PEG10 may play a specific role in mediating resistance to CDK4/6 inhibitors. Cell line details and IC_50_ data are provided in the Supplementary Table S3.

Our previous study demonstrated that expression of EMT markers, including ZEB1, N-cadherin, and vimentin was upregulated in palbociclib-resistant cells, suggesting the potential association between EMT and CDK4/6 inhibitor resistance [6], whether EMT has a causal or bystander effect. In addition, PEG10 was reported to be involved in the EMT pathway [18]. We first shortlisted 23 known EMT-associated genes from previous studies [34–36]. The heat map represented the expression level of those 23 genes in our microarray data sets, suggesting EMT process activation (Fig. 1F). Then, of 23 EMT-associated genes, 6 representative genes such as ZEB1, E-cadherin, N-cadherin, vimentin, SNAIL, and LAMC2 were further validated with qRT-PCR; those 6 representative genes were significantly upregulated in palbociclib-resistant cells, compared with their corresponding parental cells (Fig. 1G). Moreover, palbociclib-resistant cells showed mesenchymal morphology compared with their corresponding parental cells (Supplementary Fig. S1A). Next, western blot analysis supported the EMT process activation by showing the increased ZEB1 and LAMC2 but decreased E-cadherin in palbociclib-resistant cells (Fig. 1H), even though couple of genes did not show the similar pattern to that of RNA expression probably due to post-transcriptional modification. In addition, we analyzed the GDSC database to evaluate the association of EMT-associated genes (e.g., ZEB1, N-cadherin, TWIST) with palbociclib IC_50_. We found a trend toward a higher expression of mesenchymal markers such as ZEB1, N-cadherin, and TWIST, and lower expression of epithelial marker E-cadherin (Fig. 1I and Supplementary Fig. S1B) in cells with lower palbociclib activity (IC_50_ > 3.5 μM) among 11 HR+ breast cancer cell lines. These data suggest that the EMT is involved in resistance to CDK4/6 inhibitors.

### Ectopic overexpression of PEG10 augments EMT and induces palbociclib resistance

**Fig. 2:**
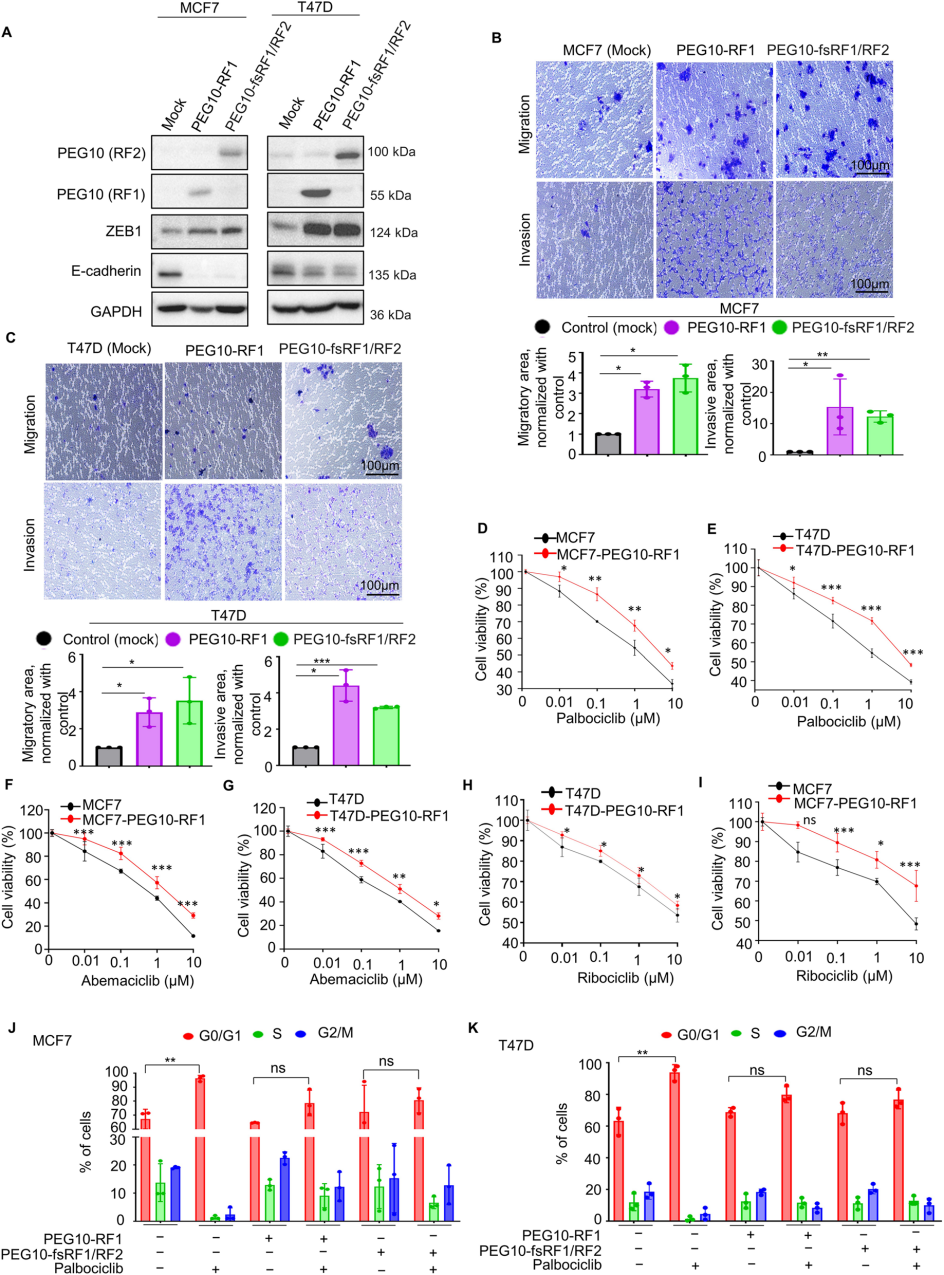
Ectopic overexpression of PEG10 augments EMT and leads to palbociclib resistance. **A** Immunoblot demonstrates ectopic overexpression of different PEG10 protein isoforms in MCF7 and T47D cells. Ectopic overexpression of PEG10 elevated the ZEB1 and suppressed the E-cadherin expression. **B**, **C** Representative images from a migration and invasion assay after ectopic overexpression of different PEG10 protein isoforms in MCF7 and T47D cells. The area of migratory and invading cells from three different non-overlapping 100 × microscopic fields is expressed as mean ± SD in the right panel. Independent sample t-test: *p < 0.05, **p < 0.01, ***p < 0.001. **D**-**I** Cell viability (MTT) assay of MCF7 and T47D cells before and after ectopic overexpression of PEG10-RF1 and subsequent treatment with the indicated dose of palbociclib, abemaciclib, and ribociclib for 72 h. Three independently repeated experiments were performed with similar results. Independent sample t-test: *p < 0.05, **p < 0.01, ***p < 0.001, Abbreviation: ns, not significant. **J**, **K** Cell cycle distribution of MCF7 and T47D cell line before and after the ectopic overexpression of different PEG10 protein isoforms and subsequent treatment with the IC_50_ concentration of palbociclib for 48 h. Three independently repeated experiments were performed with similar results. Independent sample t-test: **p < 0.01, Abbreviation: ns, not significant

Based on our results that expression of PEG10 and the EMT-related genes was upregulated in the palbociclib-resistant cells, we sought to determine whether PEG10 affects the EMT pathway. Here we ectopically overexpressed the different PEG10 protein isoforms, such as PEG10-RF1, to identify the function of the RF1 protein and frameshift mutant PEG10-fsRF1/RF2 to uncover the role of RF2 protein. Schematic structures of the PEG10 protein isoforms are shown in Supplementary Fig. S1C. Intriguingly, ectopic overexpression of PEG10 in MCF7 and T47D cells remarkably increased the ZEB1 expression but decreased the E-cadherin expression (Fig. 2A), suggesting the gain of EMT characteristics. As EMT phenotype is known to be associated with the acquisition of migratory and invasive ability of cells [37, 38], we conducted a functional assay such as migration and invasion assay after ectopic overexpression of PEG10 in MCF7 and T47D cells. As expected, PEG10-overexpressing cells exhibited a significant increase in migratory and invasive ability, compared with those in parental cells (Fig. 2B and C). This finding suggests that PEG10 may activate the EMT process.

We then examined whether the ectopic overexpression of PEG10 induces palbociclib resistance. We performed proliferation assays using PEG10-overexpressing cells with different isoforms. As expected, overexpression of PEG10 significantly decreased the palbociclib sensitivity compared with that in the parental cells (MCF7 and T47D) (Fig. 2D–I). Moreover, we performed drug sensitivity testing using other CDK4/6 inhibitors, such as abemaciclib and ribociclib, to show significantly decreased drug sensitivity in PEG10-overexpressing MCF7 and T47D cells (Supplementary Fig. S2A–F).

CDK4/6 inhibitors suppress the phosphorylation of RB, leading to G1 arrest of the cell cycle [5]. Therefore, to evaluate how PEG10 affects cell cycle, we overexpressed the PEG10-RF1 and PEG10-fsRF1/RF2 in palbociclib-sensitive MCF7 and T47D cells. Treatment of palbociclib did not increase G1 arrest of both PEG10-overexpressing cells with PEG10-RF1 and PEG10-fsRF1/RF2 (Fig. 2J and K). FlowJo cell cycle raw data are presented in Supplementary Fig. S3A and B. These results suggest that overexpression of PEG10 promotes CDK4/6 inhibitor resistance in breast cancer cells by preventing G1 arrest of the cell cycle.

### PEG10 inhibition suppresses EMT and overcomes palbociclib resistance

**Fig. 3:**
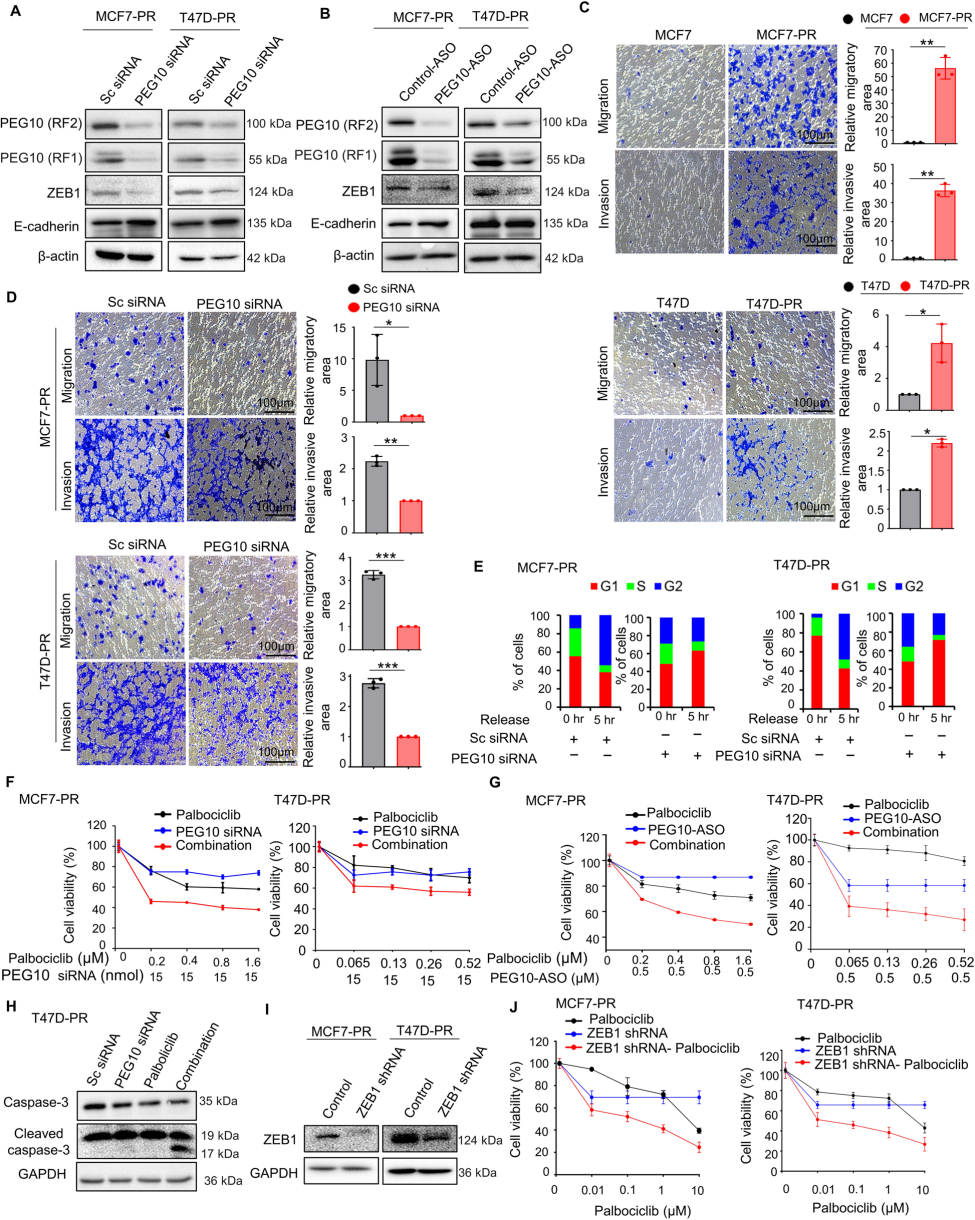
PEG10 inhibition suppresses EMT and overcomes palbociclib resistance. **A**, **B** Immunoblot showed changes in the ZEB1 and E-cadherin expression after the knockdown of PEG10 using PEG10 siRNA and PEG10-ASO in MCF7-PR and T47D-PR cells. **C** Representative images from a migration and invasion assay of parental (MCF7 and T47D) versus palbociclib-resistant cells (MCF7-PR and T47D-PR), respectively. The area of migratory and invading cells from three different non-overlapping 100 × microscopic fields is expressed as mean ± SD in the right panel. Independent sample t-test: *p < 0.05, **p < 0.01, ***p < 0.001. **D** Representative image from a migration and invasion assay of palbociclib-resistant (MCF7-PR and T47D-PR) cells after the transient PEG10 knockdown by PEG10 siRNA. The area of migratory and invading cells from three different non-overlapping 100 × microscopic fields is expressed as mean ± SD in the right panel. Independent sample t-test: *p < 0.05, **p < 0.01, ***p < 0.001. **E** Cell cycle analysis using PI staining after PEG10 knockdown in MCF7-PR and T47D-PR cells. The cell cycle was initially synchronized (syn) at G0/G1 with a double-thymidine block and then released and analyzed at the indicated time points. The bar represents the cell population distribution in each phase of the cell cycle. **F** Cell viability (MTT) assay of MCF7-PR and T47D-PR cells after treatment with PEG10 siRNA or palbociclib and combination of various concentrations of palbociclib and a fixed concentration of siRNA for 72 h. Three independently repeated experiments were performed with similar results. **G** Cell viability (MTT) assay of MCF7-PR and T47D-PR cells after treatment with PEG10-ASO or palbociclib and combination of various concentrations of palbociclib and a fixed concentration of ASO for 72 h. Three independently repeated experiments were performed with similar results. **H** Immunoblot showed induction of cleaved caspase-3 in the combination treatment of PEG10 siRNA and palbociclib. **I** Immunoblot showed ZEB1 inhibition by ZEB1 shRNA for 48 h. **J** Cell viability (MTT) assay of MCF7-PR and T47D-PR cells after treatment with ZEB1 shRNA or palbociclib and combination of various concentrations of palbociclib and a fixed concentration of ZEB1 shRNA for 72 h. Three independently repeated experiments were performed with similar results

After we demonstrated that overexpression of PEG10 augments the EMT and induces palbociclib resistance, we then investigated whether PEG10 inhibition would reverse the EMT and restore palbociclib sensitivity in palbociclib-resistant cells. First, we checked the knockdown efficacy of PEG10 siRNA in MCF7-PR and T47D-PR cells, which demonstrated a marked decrease in PEG10 protein expression levels (Fig. 3A). Similarly, we assessed the in vitro knockdown efficacy of five different PEG10-ASOs in MCF7-PR, T47D-PR cells. Moreover, endogenously PEG10 expressing prostate cancer cell PC3 was used as a positive control to confirm the in vitro PEG10-ASO knockdown efficacy. PEG10-ASO 4 was an effective candidate that reduced the mRNA and protein expression level of PEG10 at the greatest amount (Supplementary Fig. S4A–D). Then, in the subsequent assays, knockdown of PEG10 by siRNA or ASO markedly decreased ZEB1 expression and increased that of E-cadherin (Fig. 3A and B). Moreover, following functional assays such as migration and invasion assay confirmed the impact of PEG10 on EMT process. That is, the migratory and invasive ability significantly increased in palbociclib-resistant cells, compared with their corresponding parental cells (Fig. 3C). On the contrary, PEG10 knockdown by siRNA in palbociclib-resistant cells decreased the migratory and invasive ability again (Fig. 3D).

We next evaluated how PEG10 inhibition affects cell cycle progression. Parental and palbociclib-resistant cells were treated with scramble and PEG10 siRNA after cell cycle synchronization via double thymidine block. PEG10 siRNA increased the G0–G1 phase arrest of the cell cycle in the palbociclib-resistant cells (Fig. 3E and Supplementary Fig. S5A and B) but not that in the palbociclib-sensitive cells (Supplementary Fig. S5C and D), accounting for how palbociclib resistance is overcome by PEG10 inhibition.

To explore whether inhibition of PEG10 synergizes with CDK4/6 inhibition, we performed a proliferation assay in MCF7-PR and T47D-PR cells after treatment with PEG10 siRNA or ASO, palbociclib, or in combination. The combined inhibition of PEG10 and CDK4/6 synergistically reduced proliferation of MCF7-PR and T47D-PR cells; (CI < 1) (Fig. 3F and G). In addition, other CDK4/6 inhibitors, such as abemaciclib or ribociclib, in combination with PEG10 inhibition showed similar synergism to palbociclib; (CI < 1) (Supplementary Fig. S6A–D). However, in palbociclib-sensitive cells (MCF7 and T47D), combined PEG10 siRNA and palbociclib did not synergistically inhibit proliferation; (CI > 1) (Supplementary Fig. S6E and F). In palbociclib-resistant cells, synergistic inhibition of proliferation with the combined PEG10 siRNA and palbociclib increased apoptosis as represented by an increase in the 17 kDa cleaved caspase-3 bands, not 19 kDa bands (Fig. 3H).

We next examined whether ZEB1 inhibition affects palbociclib sensitivity. First of all, we confirmed the knockdown efficacy of ZEB1 shRNA in the palbociclib-resistant cells (Fig. 3I) and then tested palbociclib sensitivity. Combined ZEB1 inhibition and palbociclib synergistically inhibited proliferation of palbociclib-resistant cells (MCF7-PR and T47D-PR) (Fig. 3J). This implies that one of mechanisms whereby combined inhibition of PEG10 and CDK4/6 overcomes CDK4/6 inhibitors resistance is through ZEB1, a master regulator of the EMT pathway.

### PEG10 suppresses natural cell cycle inhibitor p21 and EMT process inhibitor SIAH1

**Fig. 4:**
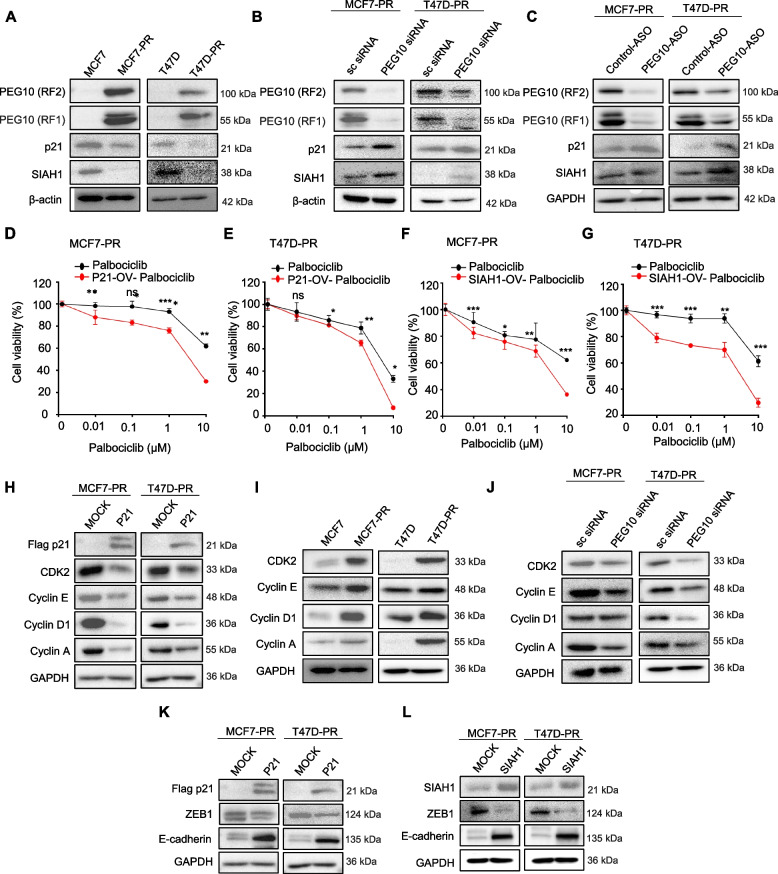
PEG10 suppresses natural cell cycle inhibitor p21 and EMT process inhibitor SIAH1. **A** Immunoblot showed changes in the cell cycle-related protein p21 and ubiquitin-related protein SIAH1 expression in parental (MCF7 and T47D) versus palbociclib-resistant (MCF7-PR and T47D-PR) cells. **B**, **C** Immunoblot showed p21 and SIAH1 protein expression level after the transient PEG10 knockdown by using PEG10 siRNA or PEG10-ASO. **D**, **E** Cell viability (MTT) assay of MCF7-PR and T47D-PR cells before and after ectopic overexpression of p21 and subsequent treatment with the various concentrations of palbociclib for 72 h. Three independently repeated experiments were performed with similar results. Independent sample t-test: *p < 0.05, **p < 0.01, ***p < 0.001, Abbreviation: ns, not significant. **F**, **G** Cell viability (MTT) assay of MCF7-PR and T47D-PR cells before and after ectopic overexpression of SIAH1 and subsequent treatment with the various concentrations of palbociclib for 72 h. Three independently repeated experiments were performed with similar results. Independent sample t-test: *p < 0.05, **p < 0.01, ***p < 0.001, Abbreviation: ns, not significant. **H** Immunoblot showed the depletion of cyclins (cyclin E, cyclin D1, and cyclin A) and CDK2 after the ectopic overexpression of p21 in MCF7-PR and T47D-PR cells. **I** Immunoblot showed the differential expression of cyclins (cyclin E, cyclin D1, and cyclin A) and CDK2 in parental cells (MCF7 and T47D) versus palbociclib-resistant cells (MCF7-PR and T47D-PR), respectively. **J** Immunoblot showed the depletion of cyclins (cyclin E, cyclin D1, and cyclin A) and CDK2 after PEG10 knockdown by PEG10 siRNA in MCF7-PR and T47D-PR cells. **K**, **L** Immunoblot showed the expression of ZEB1 and E-cadherin after the ectopic overexpression of p21 and SIAH1 in MCF7-PR and T47D-PR cells

We further sought to identify mechanisms underlying the synergism of combined inhibition of PEG10 and CDK4/6. A previous study showed that PEG10 suppressively interacts with natural CDK4/6 inhibitors, such as p16 or p18 [39] and ubiquitin ligase SIAH1 [40], a post-translational degrader of ZEB1 [41]. To test whether natural CDK4/6 inhibitors and SIAH1 participate in PEG10-associated CDK4/6 inhibitor resistance pathways or PEG10-associated EMT, we first assessed the expression levels of natural CDK inhibitors p21 and SIAH1 in palbociclib-sensitive parental cells versus resistant cells. The expression of p21 and SIAH1 was downregulated in palbociclib-resistant cells compared with that in palbociclib-sensitive parental cells (Fig. 4A). By contrast, the depletion of PEG10 by siRNA or ASO markedly increased p21 and SIAH1 expression (Fig. 4B and C).

Furthermore, we assessed the role of p21 and SIAH1 in palbociclib-resistant cells because we confirmed that expression of p21 and SIAH1 protein were regulated by PEG10. To do so, we ectopically overexpressed p21 and SIAH1 in palbociclib-resistant cells and analyzed its effects on antiproliferation. As expected, the palbociclib IC_50_ decreased down from 12 µM to 3.8 µM in MCF7-PR cells (Fig. 4D) and from 8 µM to 0.6 µM in T47D-PR cells (Fig. 4E) after ectopic overexpression of p21; similarly, ectopic overexpression of SIAH1 decreased the palbociclib IC_50_ down from 12 µM to 2.3 µM in MCF7-PR cells (Fig. 4F) and from 8 µM to 0.7 µM in T47D-PR cells (Fig. 4G). We next investigated whether p21 would affect expression of cell cycle-related genes using p21-ectopically overexpressed MCF7-PR and T47D-PR cells. Overexpression of p21 decreased expression of cell cycle-related genes, such as CDK2, cyclin E, cyclin D1, and cyclin A (Fig. 4H). Furthermore, those cell cycle-related genes were observed upregulated in our MCF7-PR and T47D-PR cells (Fig. 4I). Then, re-downregulation of cell cycle-related genes following PEG10 knockdown in MCF7-PR and T47D-PR cells confirmed that PEG10 enhances the cell cycle via suppression of p21 (Fig. 4J). Regarding the EMT process, ectopic overexpression of p21 and SIAH1 in MCF7-PR and T47D-PR cells decreased the expression of ZEB1 and increased that of E-cadherin (Fig. 4K and L), indicating EMT process suppression.

Collectively, these data suggest that PEG10 inhibits both P21 and SIAH1 in the CDK4/6 inhibitor-resistant cells. Consequently, p21 inhibition progresses the cell cycle by augmenting cyclins/CDKs, and inhibition of p21 and SIAH1 enhances the EMT process by augmenting the ZEB1. This is how PEG10 inhibition helps overcome CDK4/6 inhibitor resistance. Therefore, from the therapeutic perspective, PEG10 could be a potential therapeutic target for CDK4/6 inhibitor-resistant breast cancer.

### Combined treatment of palbociclib and PEG10-ASO regresses the palbociclib-resistant breast cancer synergistically in a xenograft model

**Fig. 5:**
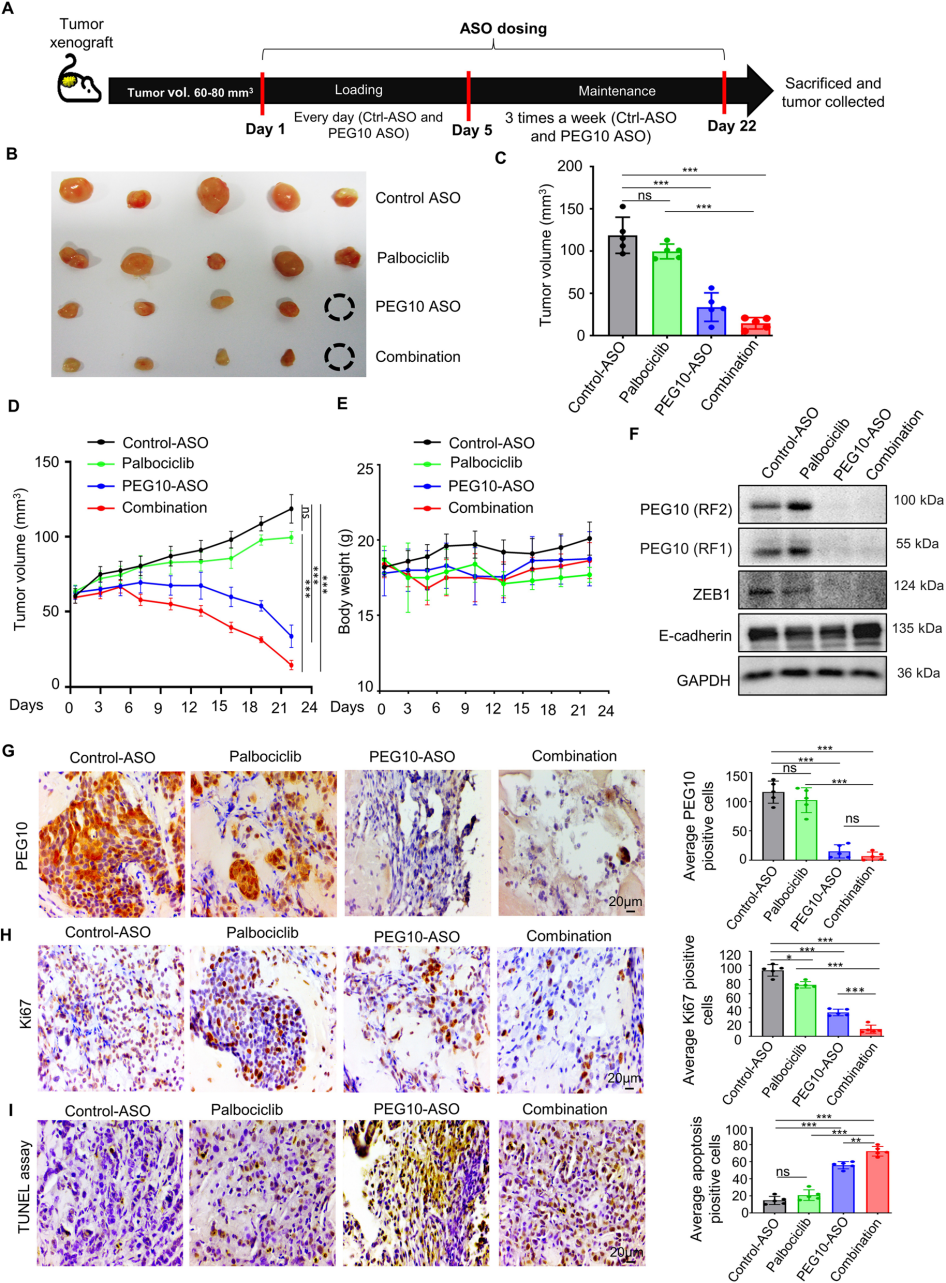
Combined treatment of palbociclib and PEG10-ASO regresses the palbociclib-resistant breast cancer synergistically in a xenograft model. **A** Experimental design for in vivo efficacy test using acquired palbociclib-resistant xenograft model in BALB/c nude mice. When tumor reached 60–80 mm3, mice were randomized to control-ASO (*n* = 5), palbociclib (*n* = 5), PEG10-ASO (*n* = 5), and combination (*n* = 5) treatment groups. Control-ASO and PEG10-ASO were administrated intraperitoneally (i.p.) for 3 weeks (15 mg/kg/day for first 5 days loading, followed by 17 days of maintenance dose of 15 mg/kg, 3 times/week). Palbociclib (100 mg/kg/day) was given by oral gavage for 3 weeks. **B** Gross harvested tumor. The black dotted circles represent the complete regression of the tumor. **C** Average tumor sizes of the indicated treatment groups before sacrifice. Independent sample t-test: ***p < 0.001, Abbreviation: ns, not significant. **D** Average tumor growth of MCF7-PR xenograft tumor treated with indicated drugs. Error bars represent SD of 5 tumors per group. Independent sample t-test: ***p < 0.001, Abbreviation: ns, not significant. **E** Average body weight of mice with indicated groups. Error bars represent the SD of 5 mice per group. **F** Immunoblots using xenograft tumors showed the suppression of PEG10 in the PEG10-ASO and combination treatment groups, along with altered ZEB1 and E-cadherin expression. **G** IHC staining of PEG10 in MCF7-PR xenograft tumors of indicated groups. Staining images were taken at 400 × magnification. The bar graph represented the PEG10 protein expression in five random, non-overlapped fields. Data are presented as mean ± SD. Independent sample t-test: ***p < 0.001, Abbreviation: ns, not significant. **H** IHC staining of Ki67 in MCF7-PR xenograft tumors of indicated groups. Staining images were taken at 400 × magnification. The bar graph represented the Ki67 cells in five random, non-overlapped fields. Data are presented as mean ± SD. Independent sample t-test: *p < 0.05, ***p < 0.001. **I** TUNEL assay using xenograft tumors at sacrifice. Staining images in each group are shown and bar graphs represent average apoptotic cells in each group in five random, non-overlapped fields at 400 × magnification. Data are presented as mean ± SD. Independent sample t-test: **p < 0.01, ***p < 0.001, Abbreviation: ns, not significant

Based on these findings, we next sought to determine whether targeting PEG10 with ASO or in combination with palbociclib could suppress tumor growth. First, the palbociclib-resistant breast cancer xenograft model was established using MCF7-PR cells. As shown in Fig. 5A, when tumors reached 60–80 mm^3^, control-ASO or PEG10-ASO was administered every day for 5 days as a loading dose, then control-ASO or PEG10-ASO was administered three times a week while palbociclib was administered every day for a total of 3 weeks. Finally, mice were euthanized, and tumors were excised 22 days after drug treatment (Fig. 5B).

Significant tumor regression was observed in groups of PEG10-ASO alone (*p* < 0.001) or PEG10-ASO and palbociclib combination compared with that treated with palbociclib alone (*p* < 0.001) or the control-ASO (Fig. 5C). In particular, the combination group exhibited a greater degree of tumor regression than that in the PEG10-ASO alone group, which parallels the in vitro results. Palbociclib treatment did not induce significant tumor regression compared with that observed with the no-treatment control (i.e., Control-ASO) (Fig. 5D), which indicates that the xenograft model was palbociclib-resistant. Importantly, none of the treatments caused weight loss, indicating a lack of generalized toxicity (Fig. 5E). Next, xenograft tumor tissues were analyzed using various assays. Significant PEG10 suppression with PEG10-ASO in tumor samples was demonstrated using western blotting (Fig. 5F) and IHC (*p* < 0.001 between control and PEG10-ASO or combination group) (Fig. 5G). Similarly, Fig. 5F showed downregulation of ZEB1, whereas E-cadherin was upregulated in combination groups, indicating suppression of the EMT process. Xenograft tumor tissues were analyzed to evaluate proliferation and apoptosis of palbociclib-resistant cells as follows; IHC for Ki67 demonstrated decreased proliferation of cells in PEG10-ASO (*p* < 0.001) or combination group (*p* < 0.001) compared with control (Fig. 5H). The TUNEL assay demonstrated increased apoptosis in PEG10-ASO (*p* < 0.001) or combination group (*p* < 0.001) compared with control (Fig. 5I). IHC for Ki67 and TUNEL assays showed most profound effect in the combination group followed by that in the PEG10-ASO group, further supporting the results of tumor growth inhibition.

### PEG10 overexpression is associated with disease recurrence and CDK4/6 inhibitor resistance in breast cancer patients

**Fig. 6:**
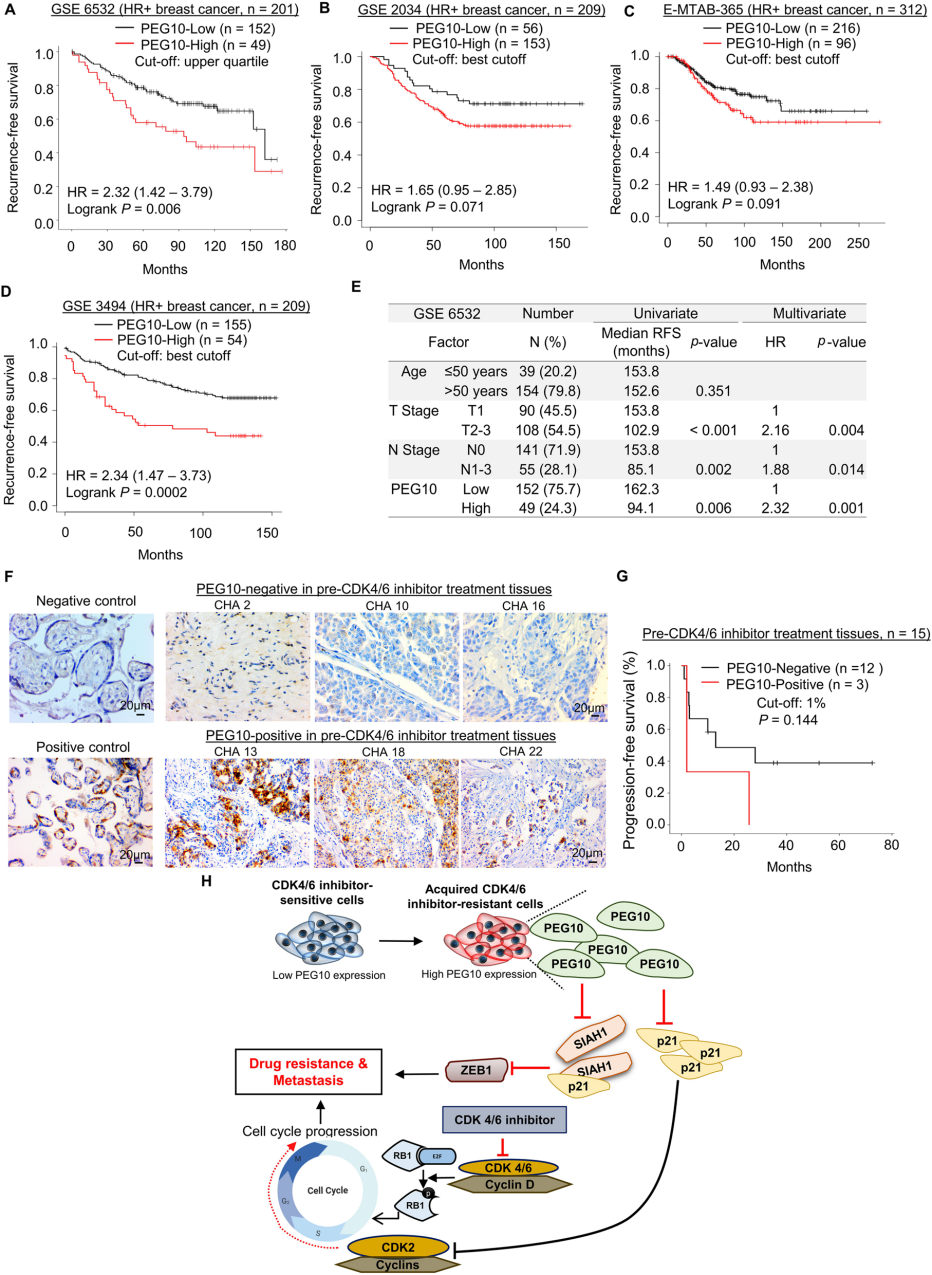
PEG10 overexpression is associated with disease recurrence and CDK4/6 inhibitor resistance in breast cancer patients. **A** Kaplan–Meier survival curves of RFS in HR+ breast cancer according to relative PEG10 mRNA expression from public mRNA microarray data set of GSE 6532. GSE 6532 analysis was performed using self-downloaded processed data. **B**-**D** Kaplan–Meier survival curves of RFS in HR+ breast cancer according to relative PEG10 mRNA expression from public mRNA microarray data sets of **B** GSE 2034, **C** E-MTAB-365, and **D** GSE 3494. Analyses of these data sets were performed using online platform Kaplan–Meier plotter (https://kmplot.com/analysis/). **E** Univariate and multivariate analysis of prognosticators for RFS in GSE 6532. **F** IHC staining of PEG10 in patients’ tumor specimens. Negative control (placental tissue, without primary antibody) and positive control (placental tissue, with primary antibody) are shown in the left panel. Representative images of negative and positive PEG10 IHC in pre-CDK4/6 inhibitor treatment tissue are shown in the right panel. **G** Kaplan–Meier survival curves of PFS according to PEG10 expression by IHC in pre-CDK4/6 inhibitor treatment tissue. **H** Schematic diagram showing the proposed mechanism that explains how PEG10 is associated with CDK4/6 inhibitors resistance. Abbreviations: RFS, recurrence-free survival; HR (in table of figure), hazard ratio; PFS, progression-free survival; Cut-off for PEG10-High versus PEG10-Low is the upper quartile

We investigated the impact of PEG10 expression on prognosis using independent public mRNA expression data sets. As previously mentioned in the methods section, to determine on optimal cut-off point for PEG10-High and PEG10-Low groups, we used minimum *p*-value approach in predicting RFS [33]. The cut-offs are set at upper quartile (75%) for GSE 6532 and auto-select best cut-off for GSE 2034, E-MTAB-365, GSE 3494, and GSE 25066. The patients with high expression level of PEG10 (‘PEG10-High’ group) had a shorter RFS than patients with low expression level of PEG10 (‘PEG10-Low’ group) in 4 of 5 GSE data sets of patients with HR+ early breast cancer (Fig. 6A-D), even though the analysis of GSE 25066 did not support our data (Supplementary Fig. S7). Furthermore, in the GSE 6532 data set, multivariate analysis, adjusted by T and N stage showed that the PEG10-High status (cut-off for PEG10-High versus PEG10-Low is the upper quartile) was a significant poor prognostic factor for recurrence in patients with HR+ early breast cancer [hazard ratio (HR) = 2.32, *p* = 0.001] (Fig. 6E). Overall, clinical relevance of PEG10 as a therapeutic target was supported by a significant association between PEG10 expression and poor prognosis based on these public mRNA databases. In addition, PEG10 IHC was performed in the 15 pre-CDK4/6 inhibitor treatment tissues from patients who received CDK4/6 inhibitor as a first-line therapy (Fig. 6F). PEG10 positivity rate was 20% (3 of 15 patients). PEG10-positive group showed a trend toward shorter CDK4/6 inhibitor PFS than PEG10-negative group (Fig. 6G; *p* = 0.144), indicating that PEG10 protein level in the tumor sample may serve as a novel biomarker to predict palbociclib efficacy.

As illustrated schematically in Fig. 6H, we suggest that PEG10 suppresses both p21, a natural CDK inhibitor and SIAH1, a post-translational degrader of ZEB1 in CDK4/6 inhibitor-resistant cells. Thus, PEG10-induced cell cycle progression and PEG10-induced EMT activation account for PEG10-associated mechanism for CDK4/6 inhibitor resistance. Consequently, combined PEG10 inhibition and palbociclib overcomes CDK4/6 inhibitor resistance.

## Discussion

CDK4/6 inhibitor in combination with endocrine therapy is highly effective treatment for HR+ breast cancer. However, acquired resistance is inevitable in most cases. Understanding mechanisms of acquired resistance and developing new strategies to overcome the resistance are critical. To investigate resistance mechanisms and overcoming strategies, we used our preclinical model of acquired palbociclib resistance, with which we had previously proved that activation of CDK2-cyclin E pathway is a mechanism of CDK4/6 inhibitor resistance [6]. In this study, we demonstrated that the high PEG10 expression is one of the mechanisms associated with acquired CDK4/6 inhibitor resistance in HR+ breast cancer. The mechanistic study confirmed that high PEG10 expression suppressed the p21, a natural CDK inhibitor, and SIAH1, a post-translational degrader of ZEB1, augmenting CDK4/6 inhibitor resistance. Then PEG10 inhibition combined with palbociclib suppressed cell cycle progression and EMT via activating p21 and SIAH1, respectively. Consequently, we proved that our in-house PEG10 inhibitor, PEG10-ASO combined with a CDK4/6 inhibitor could be a promising strategy to overcome CDK4/6 inhibitor resistance.

As mentioned above, our mechanistic study confirmed that PEG10 augments CDK4/6 inhibitor resistance by inhibiting the activity of p21 and SIAH1. In addition to several CDK4/6 inhibitor resistance mechanisms, research focusing on natural CDK inhibitor such as CDK interacting protein (CIP)/kinase inhibitory protein (KIP), p21 could be a promising approach to overcome CDK4/6 inhibitor resistance [42, 43]. For instance, a recent study indicated histone deacetylases (HDACs) mediate palbociclib resistance by downregulating the expression of p21 [44]. Accordingly, we demonstrated that synergy of palbociclib and PEG10 inhibition to overcome CDK4/6 inhibitor resistance is mediated by enhancement of the activity of p21, a natural CDK4/6 inhibitor. Meanwhile, regarding association of EMT and resistance mechanism in our study, although several studies reported that EMT activation contributed to resistance of various anticancer therapies, including asimertinib [45], chemotherapy [46, 47], and radiotherapy [48], this is the first report that EMT contributed to development of CDK4/6 inhibitor resistance. In more detail, we demonstrated that PEG10 inhibited SIAH1 and consequently increased ZEB1, a master regulator of EMT. In this study, from the therapeutic perspective, ZEB1 suppression by PEG10 inhibition attenuated EMT and could help overcome CDK4/6 inhibitor resistance. The specific effect of EMT inhibition on CDK4/6 inhibitor resistance was confirmed by shRNA-mediated knockdown of ZEB1. However, the detailed mechanisms of how EMT pathway affects resistance to various drugs, including CDK4/6 inhibitors, remain to be elucidated. Our study demonstrates that CDK4/6 inhibitor resistance could be overcome by suppressing both the CDKs/cyclins and EMT through PEG10 inhibition.

We designed an ASO drug to target PEG10 for in vivo study because a specific inhibitor of PEG10 has yet to be developed for the clinic or in preclinical research. Our PEG10-ASO in combination with palbociclib in the palbociclib-resistant xenograft model, decreased tumor size more profoundly than PEG10-ASO or palbociclib alone. ASOs act as promising therapeutic approach for a wide range of diseases. One of the major advantages of ASOs is their specificity that enables the selective targeting of disease-causing genes. ASOs may be more advantageous in cases where single genes are responsible for each disease [49]. Nusinersen (Spinraza) is a well-known ASO drug was to treat spinal muscular atrophy associated with a mutation in the SMN1 gene [50]. In addition to their specificity, ASO drugs are cheaper and easy to design and modify to enhance their stability and penetration into cells. By contrast, conventional small-molecule pharmaceuticals require much larger and often iterative screening efforts followed by extensive medicinal chemistry optimization. ASO drugs may target traditionally “undruggable” targets, including proteins that lack hydrophobic pockets. However, several disadvantages are also associated with these ASO drugs. Limited delivery of ASO drugs remains a major challenge. Recently chemical modification and bioconjugation as well as the use of nanocarriers have been tried to enhance efficiency of ASO delivery [51]. Variable unexpected adverse events may be caused by off-target effects, as well. Therefore, researchers are seeking to reduce off-target effects by modifying aspects of ASOs, such as their length [52]. Despite these disadvantages, development of ASO drugs continues to progress because of greater advantages in selective targetability and easier druggability of undruggable targets. Although no approved ASO drug currently exists for any type of cancer, the results obtained in preclinical studies and clinical trials are encouraging [53].

To assume potential toxicities of PEG10 inhibition in patients, we searched for publications regarding outcomes in PEG10-knockout mice. PEG10-knockout mice exhibited embryonic lethality by 10.5 days post coitus and those that survived past the embryonic stage exhibited growth retardation and a decreased lifespan. These results suggest that PEG10 is essential for both embryonic development and postnatal growth and development [23]. Therefore, when clinically applied, a PEG10 inhibitor should be cautiously administered to patients with child-bearing potential. We did not observe any toxicities, including weight loss, in our in vivo study, which indicates that PEG10-ASO may be well tolerated. However, further research is needed to fully understand the toxic effects of PEG10 inhibition on normal tissues.

Although many preclinical studies regarding various mechanisms of CDK4/6 inhibitor resistance have been published, only a few new drugs are in clinical trials as shown in Supplementary Table S6. Two classes of cell cycle inhibitors, CDK7 inhibitor samuraciclib [54] and CDK2/4/6 inhibitor ebvaciclib [55], have entered clinical development. In particular, samuraciclib has been granted fast-track status by the United States Food and Drug Administration in 2021, based on preliminary effects of samuraciclib plus fulvestrant in patients who were heavily pretreated with CDK4/6 inhibitor in the phase II trial [54]. Of the growth factor inhibitors, only FGFR inhibitor erdafitinib [56] and AKT inhibitor ipatasertib [57] are under clinical trial in a post-CDK4/6 inhibitor setting. As mentioned earlier, no dominant mechanism has been identified for CDK4/6 inhibitor resistance. Therefore, we suggest that high PEG10 expression is responsible for a CDK4/6 inhibitors resistance in a portion of patients, and our study provides the rationale for future development of PEG10 inhibitor.

The limitation of this study is that we used only small number of patient specimens, retrospectively collected to determine the impact of PEG10 expression on the outcome of CDK4/6 inhibitor therapy. Further confirmation is needed with analysis using larger size of patient’s data.

## Conclusions

Collectively, our study suggests that PEG10 overexpression is a novel mechanism behind CDK4/6 inhibitor resistance through cell cycle progression and EMT activation. We also validated PEG10 as a promising therapeutic target. To date, there are no PEG10 inhibiting drugs available. Therefore, the development of a PEG10 inhibitor is a rational approach for the treatment of breast cancer with PEG10-associated resistance to CDK4/6 inhibitor.

### Supplementary Information

**Additional file 1:** **Fig. S1.** (A) Images showing the morphological alternations in the acquired palbociclib-resistant cells (MCF7-PR and T47D-PR) compared with the parental (MCF7 and T47D) cells. Scale bar = 100 µm. (B) Association of E-cadherin, N-cadherin, and TWIST genes and palbociclib sensitivity in 11 HR+ breast cancer cell lines from GDSC database. Palbociclib sensitivity was defined as IC_50_ ≤ 3.5 µM. *P-*value was calculated by independent sample t-test. (C) Figure showing PEG10 protein isoforms used in this study. ORF1 and ORF2 are colored black and separated by green marked overlapping region, the purple marking indicates the frameshift site and red markings show the termination codons of the translation, while blue color highlights active site motif.

**Additional file 2:** **Fig. S2.** (A-C) Cell viability (MTT) assay of MCF7 cell line before and after ectopic overexpression of PEG10-fsRF1/RF1 and subsequent treatment with the various concentrations of palbociclib, abemaciclib, and ribociclib for 72 h. Three independently repeated experiments were performed with similar results. Independent sample t-test: *p < 0.05, **p < 0.01, ***p < 0.001, Abbreviation: ns, not significant. (D-F) Cell viability (MTT) assay of T47D cell line before and after ectopic overexpression of PEG10-fsRF1/RF1 and subsequent treatment with the various concentrations of palbociclib, abemaciclib, and ribociclib for 72 h. Three independently repeated experiments were performed with similar results. Independent sample t-test: *p < 0.05, **p < 0.01, ***p < 0.001, Abbreviation: ns, not significant.

**Additional file 3:** **Fig. S3.** (A-B) Data underlying the plots in (Fig. 2J and K), showing flowJo cell cycle analysis using PI staining after ectopic overexpression of PEG10 isoforms and subsequent treatment with palbociclib IC_50_ for 48h.

**Additional file 4:** **Fig. S4.** (A-C) Immunoblots showed PEG10 knockdown efficacy by various PEG10-ASOs in MCF7-PR, T47D-PR, and PC3 cells. (D) qRT-PCR data showed the PEG10 knockdown efficacy by indicated PEG10-ASOs in MCF7-PR cells.

**Additional file 5:** **Fig. S5.** (A-B) Data underlying the plots in (Fig. 3E), showing flowJo cell cycle analysis using PI staining after PEG10 knockdown. (C-D) Cell cycle analysis using PI staining after PEG10 knockdown, indicating no alternation in cell cycle progression in palbociclib-sensitive MCF7 cells. The cell cycle was initially synchronized at G0/G1 with a double-thymidine block and then released and analyzed at the indicated time points. The bar represents the distribution of the cell population in each phase of the cell cycle. The right panel indicates the cell cycle analysis by flowJo.

**Additional file 6:** **Fig. S6.** (A) Cell viability (MTT) assay of MCF7-PR cells after treatment with PEG10 siRNA or abemaciclib and combination of various concentrations of abemaciclib and a fixed concentration of siRNA for 72 h. Three independently repeated experiments were performed with similar results. The CI values were calculated using the Chou–Talalay method. CI < 1 indicates synergism. (B) Cell viability (MTT) assay of MCF7-PR cells after treatment with PEG10 siRNA or ribociclib and combination of various concentrations of ribociclib and a fixed concentration of siRNA for 72 h. Three independently repeated experiments were performed with similar results. The CI values were calculated using the Chou–Talalay method. CI < 1 indicates synergism. (C) Cell viability (MTT) assay of T47D-PR cells after treatment with PEG10 siRNA or abemaciclib and combination of various concentrations of abemaciclib and a fixed concentration of siRNA for 72 h. Three independently repeated experiments were performed with similar results. The CI values were calculated using the Chou–Talalay method. CI < 1 indicates synergism. (D) Cell viability (MTT) assay of T47D-PR cells after treatment with PEG10 siRNA or ribociclib and combination of various concentrations of ribociclib and a fixed concentration of siRNA for 72 h. Three independently repeated experiments were performed with similar results. The CI values were calculated using the Chou–Talalay method. CI < 1 indicates synergism. (E-F) MTT assay of MCF7 and T47D cell lines after treatment with PEG10 siRNA or palbociclib and combination of various concentrations of palbociclib and a fixed concentration of siRNA for 72 h. Three independently repeated experiments were performed with similar results. The CI values were calculated using the Chou–Talalay method. CI > 1 and CI = 1 indicate synergism and additive effect, respectively.

**Additional file 7:** **Fig. S7.** (A) Kaplan-Meier survival curves of RFS in HR+ breast cancer according to relative PEG10 mRNA expression from public mRNA microarray data sets of GSE 25066. Analysis of this data set was performed using the online platform Kaplan-Meier plotter (https://kmplot.com/analysis/).

**Additional file 8:** **Table S1.** List of primers used for qRT-PCR. **Table S2.** List of Primary and secondary antibodies used for western blot and immunohistochemistry. **Table S3.** list of 43 breast cancer cell lines in the GDSC database focusing on general subtypes and palbociclib IC_50_. **Table S4.** Clinical characteristics of patients whose tumors were used for PEG10 IHC. **Table S5.** List of the commonly upregulated genes in palbociclib-resistant cells compared with parental cell lines. **Table S6.** New drugs under clinical trials in the setting of CDK4/6 inhibitor resistance.

## Data Availability

All the data supporting the findings of this study are available within the paper and its Supplementary Information files. All other data supporting the findings of this study are available from the corresponding author upon reasonable request.

## Abbreviations

PEG10: Paternally expressed gene 10
CDK4/6: Cyclin–dependent kinase 4 and 6
HR+: Hormone receptor–positive
EMT: Epithelial–mesenchymal transition
ASO: Antisense oligonucleotide
HER2+: Human epidermal growth factor receptor 2-positive
CDK: Cyclin–dependent kinase
RB: Retinoblastoma
qRT-PCR: Quantitative real-time polymerase chain reaction
GDSC: Genomics of drug sensitivity in cancer
ORF: Open reading frame
IC_50_: Half the maximal inhibitory concentration
MTT: 3-[4,5-Dimethylthiazol-2-yl]-2,5 diphenyltetrazolium bromide
FACS: Fluorescence-activated cell sorting
IHC: Immunohistochemistry
PFS: Progression-free survival
TUNEL: Terminal deoxynucleotidyl transferase dUTP nick end labeling
ER+: Estrogen receptor–positive
RFS: Recurrence-free survival
DEG: Differentially expressed gene
CI: Combination Index
HR: Hazard ratio
CIP/KIP: CDK interacting protein/kinase inhibitory protein
HDAC: Histone deacetylase
IACUC: Institutional animal care and use committee
