# Supplementary material for: Targeting PEG10 as a novel therapeutic approach to overcome CDK4/6 inhibitor resistance in breast cancer
Source: J Exp Clin Cancer Res. 2023 Nov 28;42:325. doi: 10.1186/s13046-023-02903-x (PMC10683152; doi:10.1186/s13046-023-02903-x)
Supplement: Supplementary file 1 — Additional file 1: Fig. S1. (A) Images showing the morphological alternations in the acquired palbociclib-resistant cells (MCF7-PR and T47D-PR) compared with the parental (MCF7 and T47D) cells. Scale bar = 100 µm. (B) Association of E-cadherin, N-cadherin, and TWIST genes and palbociclib sensitivity in 11 HR+ breast cancer cell lines from GDSC database. Palbociclib sensitivity was defined as IC50 ≤ 3.5 µM. P-value was calculated by independent sample t-test. (C) Figure showing PEG10 protein isoforms used in this study. ORF1 and ORF2 are colored black and separated by green marked overlapping region, the purple marking indicates the frameshift site and red markings show the termination codons of the translation, while blue color highlights active site motif. [file 13046_2023_2903_MOESM1_ESM.docx]

**Supplementary Figure S1**


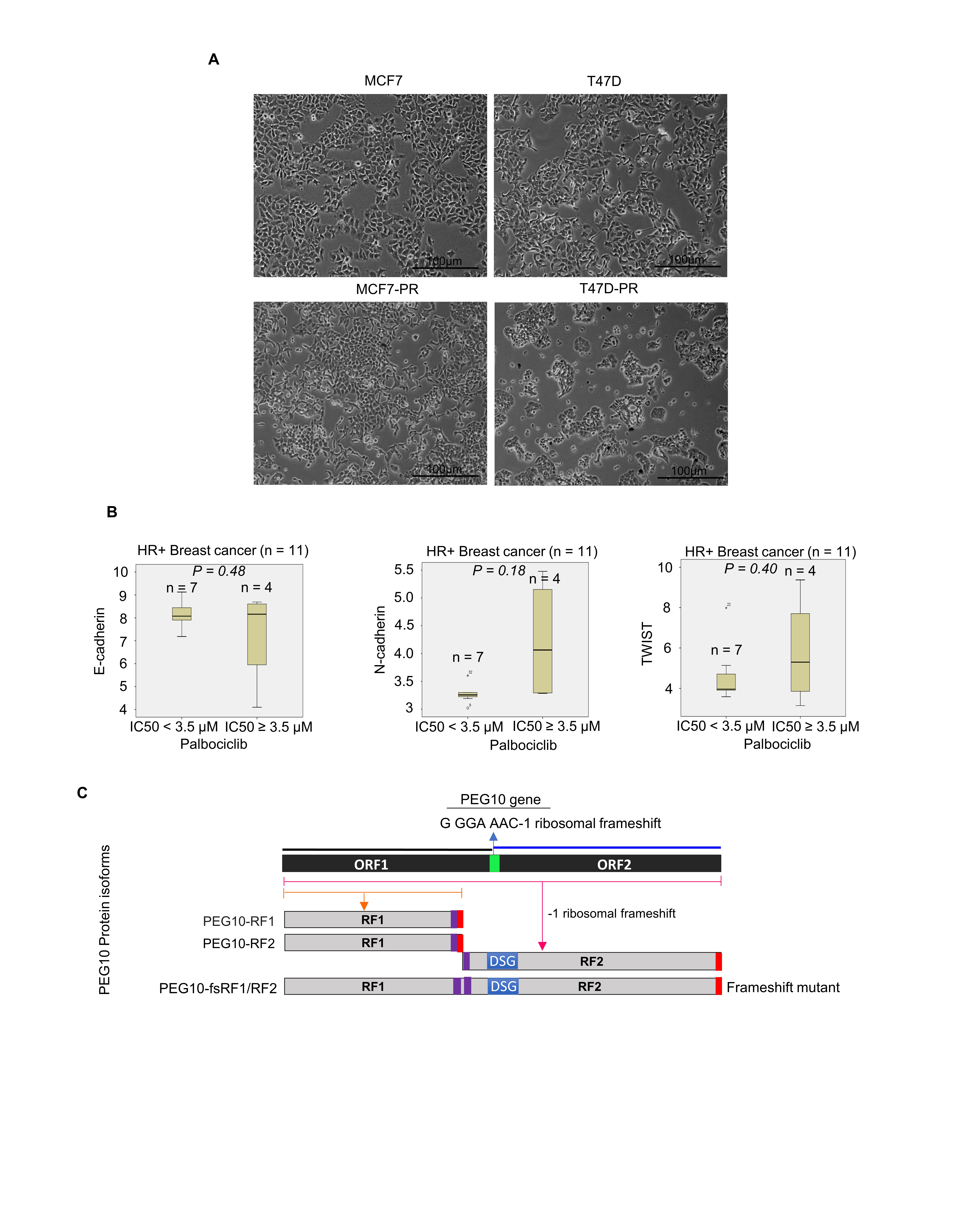


**Fig. S1.** (A) Images showing the morphological alternations in the acquired palbociclib-resistant cells (MCF7-PR and T47D-PR) compared with the parental (MCF7 and T47D) cells. Scale bar = 100 µm.

(B) Association of E-cadherin, N-cadherin, and TWIST genes and palbociclib sensitivity in 11 HR+ breast cancer cell lines from GDSC database. Palbociclib sensitivity was defined as IC_50_ ≤ 3.5 µM. *P-*value was calculated by independent sample t-test.

(C) Figure showing PEG10 protein isoforms used in this study. ORF1 and ORF2 are colored black and separated by green marked overlapping region, the purple marking indicates the frameshift site and red markings show the termination codons of the translation, while blue color highlights active site motif.
