# Supplementary material for: Targeting PEG10 as a novel therapeutic approach to overcome CDK4/6 inhibitor resistance in breast cancer
Source: J Exp Clin Cancer Res. 2023 Nov 28;42:325. doi: 10.1186/s13046-023-02903-x (PMC10683152; doi:10.1186/s13046-023-02903-x)
Supplement: Supplementary file 2 — Additional file 2: Fig. S2. (A-C) Cell viability (MTT) assay of MCF7 cell line before and after ectopic overexpression of PEG10-fsRF1/RF1 and subsequent treatment with the various concentrations of palbociclib, abemaciclib, and ribociclib for 72 h. Three independently repeated experiments were performed with similar results. Independent sample t-test: *p < 0.05, **p < 0.01, ***p < 0.001, Abbreviation: ns, not significant. (D-F) Cell viability (MTT) assay of T47D cell line before and after ectopic overexpression of PEG10-fsRF1/RF1 and subsequent treatment with the various concentrations of palbociclib, abemaciclib, and ribociclib for 72 h. Three independently repeated experiments were performed with similar results. Independent sample t-test: *p < 0.05, **p < 0.01, ***p < 0.001, Abbreviation: ns, not significant. [file 13046_2023_2903_MOESM2_ESM.docx]

**Supplementary Figure S2**


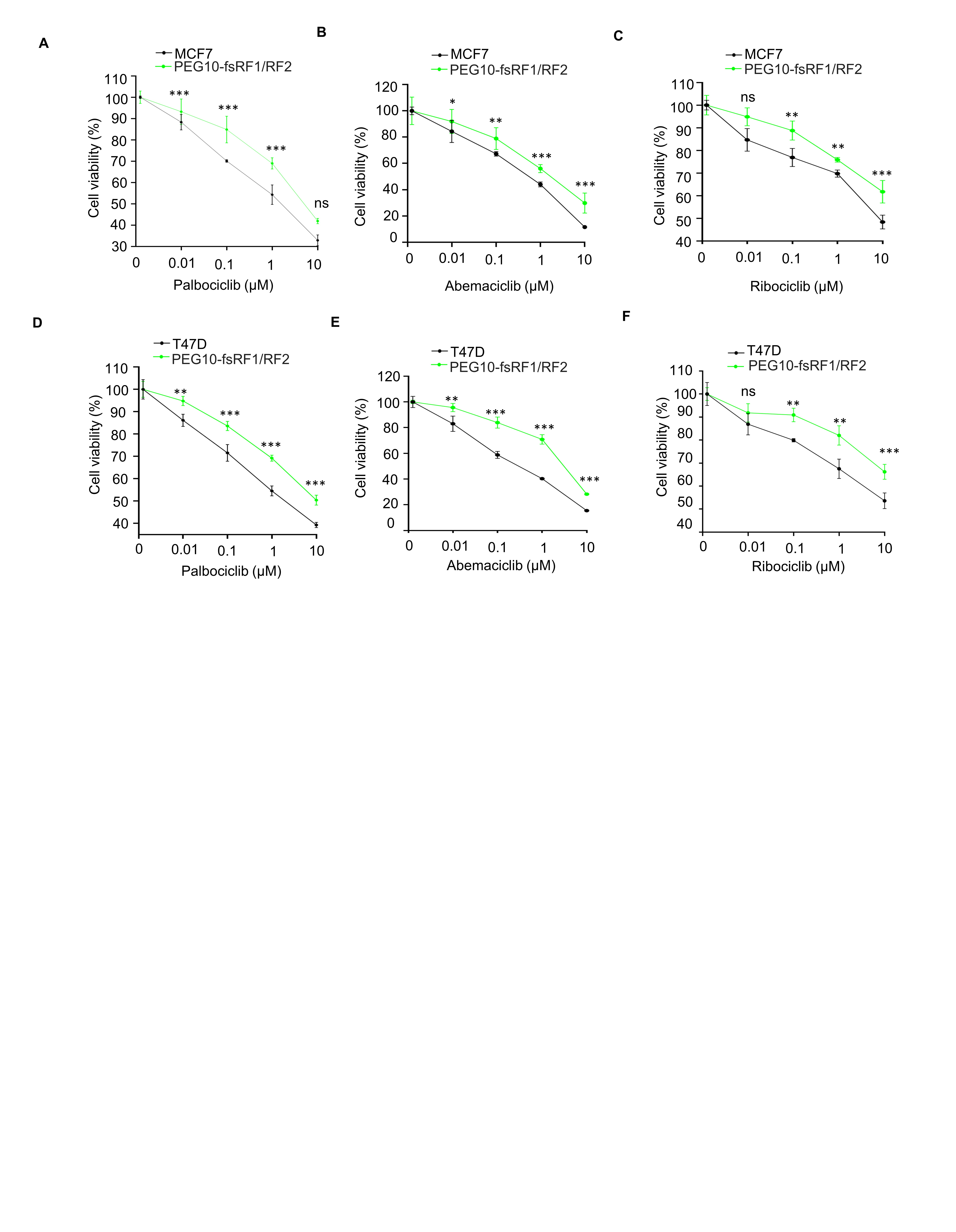


**Fig. S2.** (A-C) Cell viability (MTT) assay of MCF7 cell line before and after ectopic overexpression of PEG10-fsRF1/RF1 and subsequent treatment with the various concentrations of palbociclib, abemaciclib, and ribociclib for 72 h. Three independently repeated experiments were performed with similar results. Independent sample t-test: **p* < 0.05, ***p* < 0.01, ****p* < 0.001, Abbreviation: ns, not significant.

(D-F) Cell viability (MTT) assay of T47D cell line before and after ectopic overexpression of PEG10-fsRF1/RF1 and subsequent treatment with the various concentrations of palbociclib, abemaciclib, and ribociclib for 72 h. Three independently repeated experiments were performed with similar results. Independent sample t-test: **p* < 0.05, ***p* < 0.01, ****p* < 0.001, Abbreviation: ns, not significant.
