# Supplementary material for: Targeting PEG10 as a novel therapeutic approach to overcome CDK4/6 inhibitor resistance in breast cancer
Source: J Exp Clin Cancer Res. 2023 Nov 28;42:325. doi: 10.1186/s13046-023-02903-x (PMC10683152; doi:10.1186/s13046-023-02903-x)
Supplement: Supplementary file 3 — Additional file 3: Fig. S3. (A-B) Data underlying the plots in (Fig. 2J and K), showing flowJo cell cycle analysis using PI staining after ectopic overexpression of PEG10 isoforms and subsequent treatment with palbociclib IC50 for 48h. [file 13046_2023_2903_MOESM3_ESM.docx]

**Supplementary Figure S3**


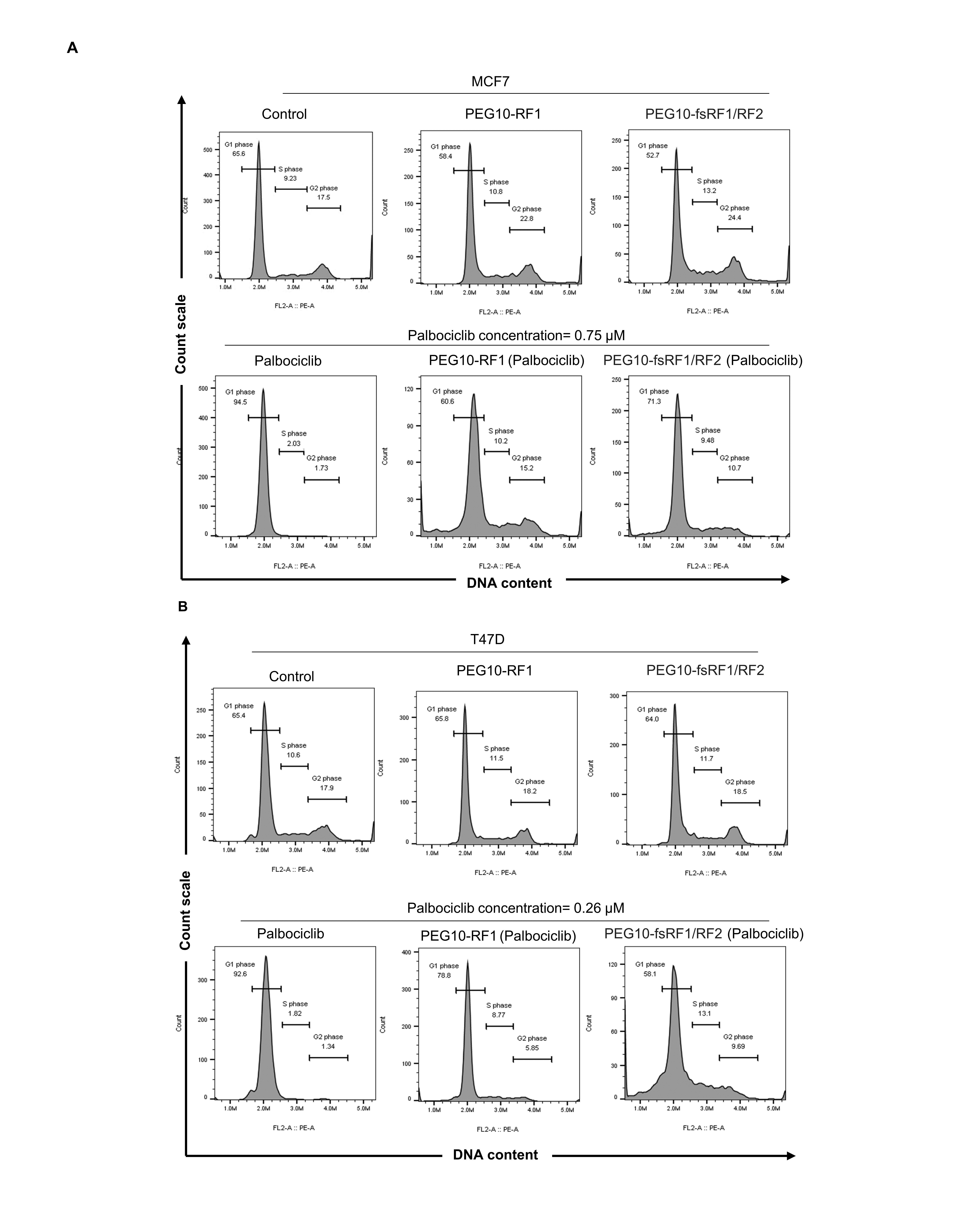


**Fig. S3.** (A-B) Data underlying the plots in (Fig. 2, J and K), showing flowJo cell cycle analysis using PI staining after ectopic overexpression of PEG10 isoforms and subsequent treatment with palbociclib IC_50_ for 48h.
