# Supplementary material for: Targeting PEG10 as a novel therapeutic approach to overcome CDK4/6 inhibitor resistance in breast cancer
Source: J Exp Clin Cancer Res. 2023 Nov 28;42:325. doi: 10.1186/s13046-023-02903-x (PMC10683152; doi:10.1186/s13046-023-02903-x)
Supplement: Supplementary file 4 — Additional file 4: Fig. S4. (A-C) Immunoblots showed PEG10 knockdown efficacy by various PEG10-ASOs in MCF7-PR, T47D-PR, and PC3 cells. (D) qRT-PCR data showed the PEG10 knockdown efficacy by indicated PEG10-ASOs in MCF7-PR cells. [file 13046_2023_2903_MOESM4_ESM.docx]

**Supplementary Figure S4**


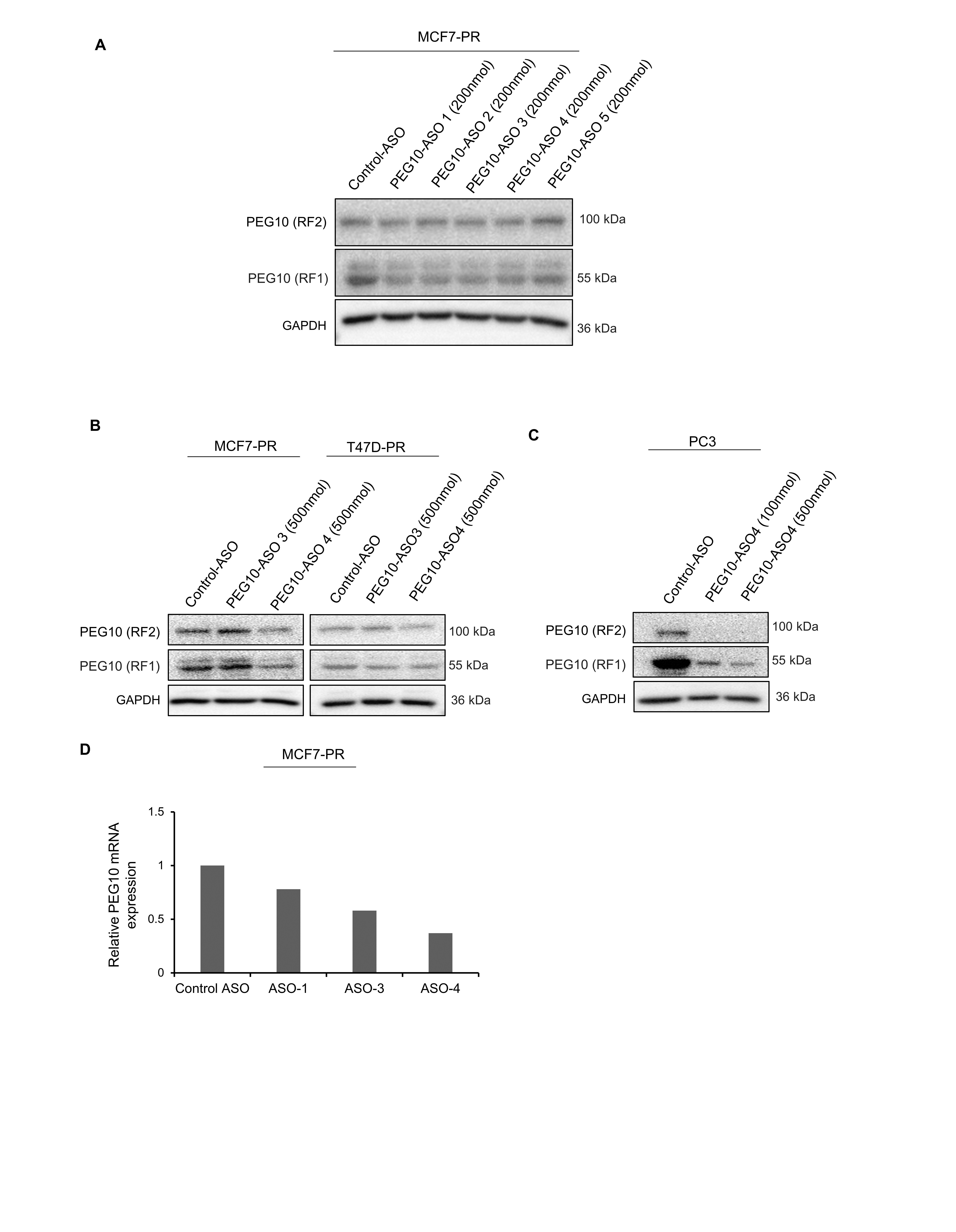


**Fig. S4.** (A-C) Immunoblots showed PEG10 knockdown efficacy by various PEG10-ASOs in MCF7-PR, T47D-PR, and PC3 cells.

(D) qRT-PCR data showed the PEG10 knockdown efficacy by indicated PEG10-ASOs in MCF7-PR cells.
