# Supplementary material for: Targeting PEG10 as a novel therapeutic approach to overcome CDK4/6 inhibitor resistance in breast cancer
Source: J Exp Clin Cancer Res. 2023 Nov 28;42:325. doi: 10.1186/s13046-023-02903-x (PMC10683152; doi:10.1186/s13046-023-02903-x)
Supplement: Supplementary file 5 — Additional file 5: Fig. S5. (A-B) Data underlying the plots in (Fig. 3E), showing flowJo cell cycle analysis using PI staining after PEG10 knockdown. (C-D) Cell cycle analysis using PI staining after PEG10 knockdown, indicating no alternation in cell cycle progression in palbociclib-sensitive MCF7 cells. The cell cycle was initially synchronized at G0/G1 with a double-thymidine block and then released and analyzed at the indicated time points. The bar represents the distribution of the cell population in each phase of the cell cycle. The right panel indicates the cell cycle analysis by flowJo. [file 13046_2023_2903_MOESM5_ESM.docx]

**Supplementary Figure S5**


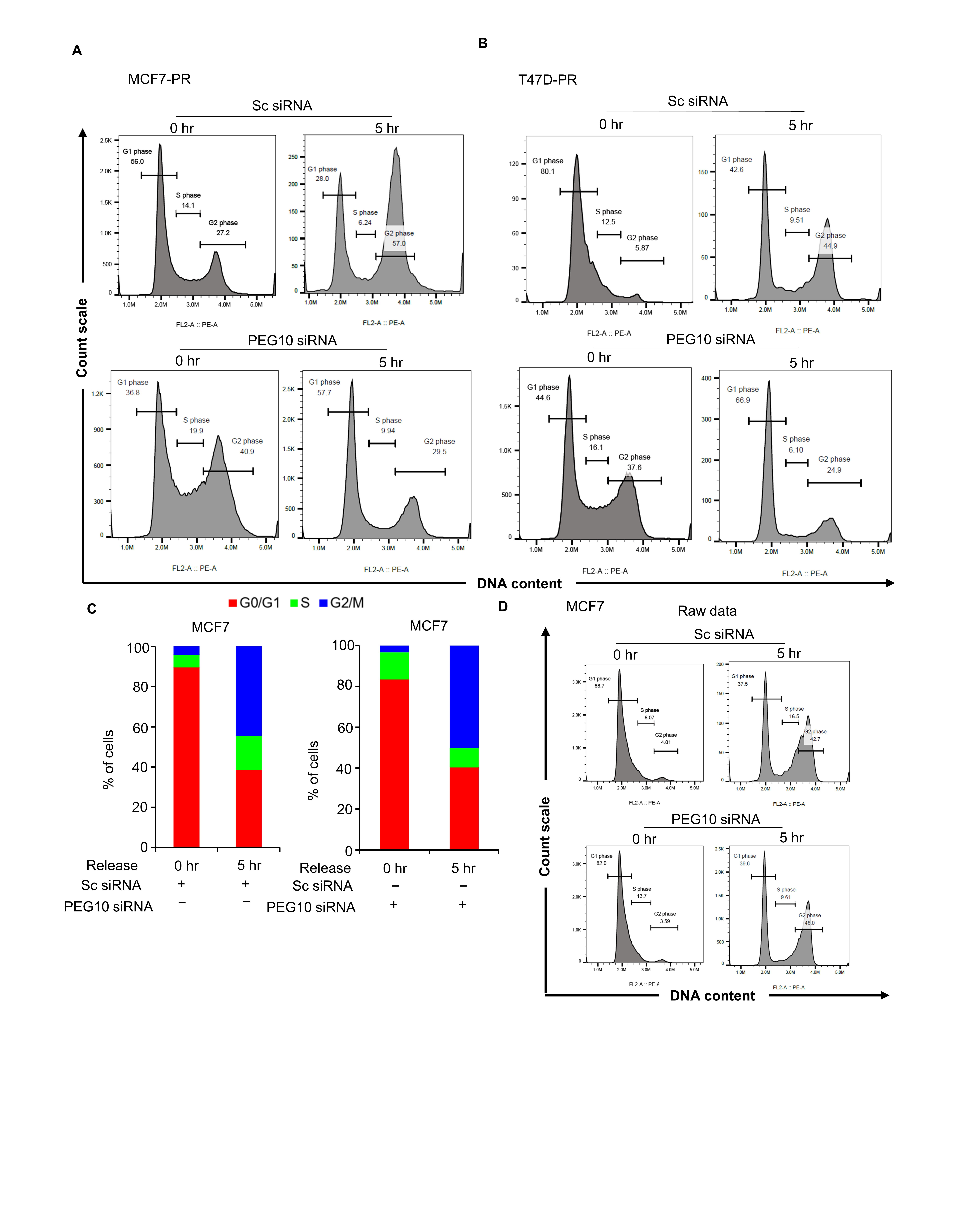


**Fig. S5.** (A-B) Data underlying the plots in (Figure 3E), showing flowJo cell cycle analysis using PI staining after PEG10 knockdown.

(C-D) Cell cycle analysis using PI staining after PEG10 knockdown, indicating no alternation in cell cycle progression in palbociclib-sensitive MCF7 cells. The cell cycle was initially synchronized at G0/G1 with a double-thymidine block and then released and analyzed at the indicated time points. The bar represents the distribution of the cell population in each phase of the cell cycle. The right panel indicates the cell cycle analysis by flowJo.
