# Supplementary material for: Targeting PEG10 as a novel therapeutic approach to overcome CDK4/6 inhibitor resistance in breast cancer
Source: J Exp Clin Cancer Res. 2023 Nov 28;42:325. doi: 10.1186/s13046-023-02903-x (PMC10683152; doi:10.1186/s13046-023-02903-x)
Supplement: Supplementary file 6 — Additional file 6: Fig. S6. (A) Cell viability (MTT) assay of MCF7-PR cells after treatment with PEG10 siRNA or abemaciclib and combination of various concentrations of abemaciclib and a fixed concentration of siRNA for 72 h. Three independently repeated experiments were performed with similar results. The CI values were calculated using the Chou–Talalay method. CI < 1 indicates synergism. (B) Cell viability (MTT) assay of MCF7-PR cells after treatment with PEG10 siRNA or ribociclib and combination of various concentrations of ribociclib and a fixed concentration of siRNA for 72 h. Three independently repeated experiments were performed with similar results. The CI values were calculated using the Chou–Talalay method. CI < 1 indicates synergism. (C) Cell viability (MTT) assay of T47D-PR cells after treatment with PEG10 siRNA or abemaciclib and combination of various concentrations of abemaciclib and a fixed concentration of siRNA for 72 h. Three independently repeated experiments were performed with similar results. The CI values were calculated using the Chou–Talalay method. CI < 1 indicates synergism. (D) Cell viability (MTT) assay of T47D-PR cells after treatment with PEG10 siRNA or ribociclib and combination of various concentrations of ribociclib and a fixed concentration of siRNA for 72 h. Three independently repeated experiments were performed with similar results. The CI values were calculated using the Chou–Talalay method. CI < 1 indicates synergism. (E-F) MTT assay of MCF7 and T47D cell lines after treatment with PEG10 siRNA or palbociclib and combination of various concentrations of palbociclib and a fixed concentration of siRNA for 72 h. Three independently repeated experiments were performed with similar results. The CI values were calculated using the Chou–Talalay method. CI > 1 and CI = 1 indicate synergism and additive effect, respectively. [file 13046_2023_2903_MOESM6_ESM.docx]

**Supplementary Figure S6**


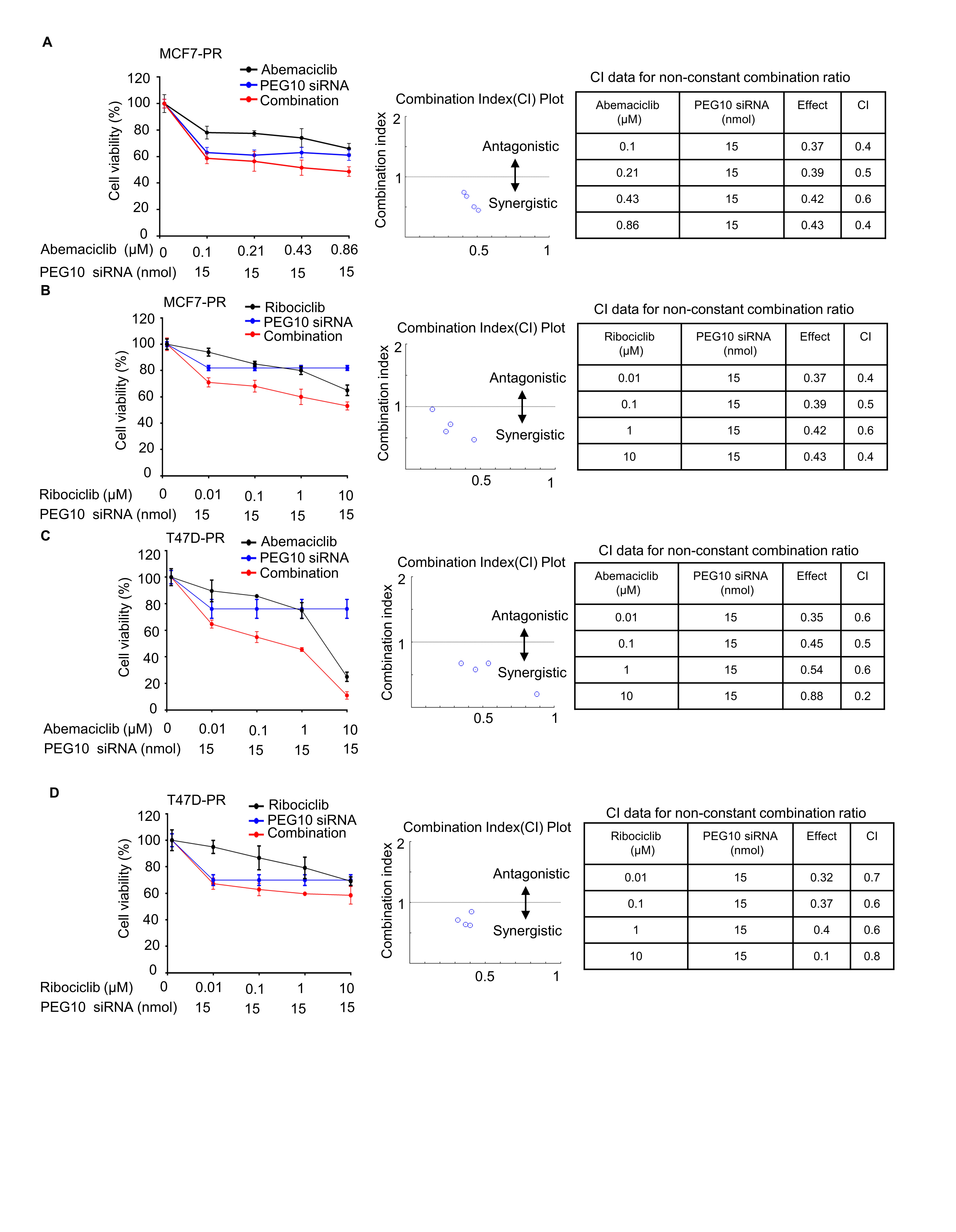


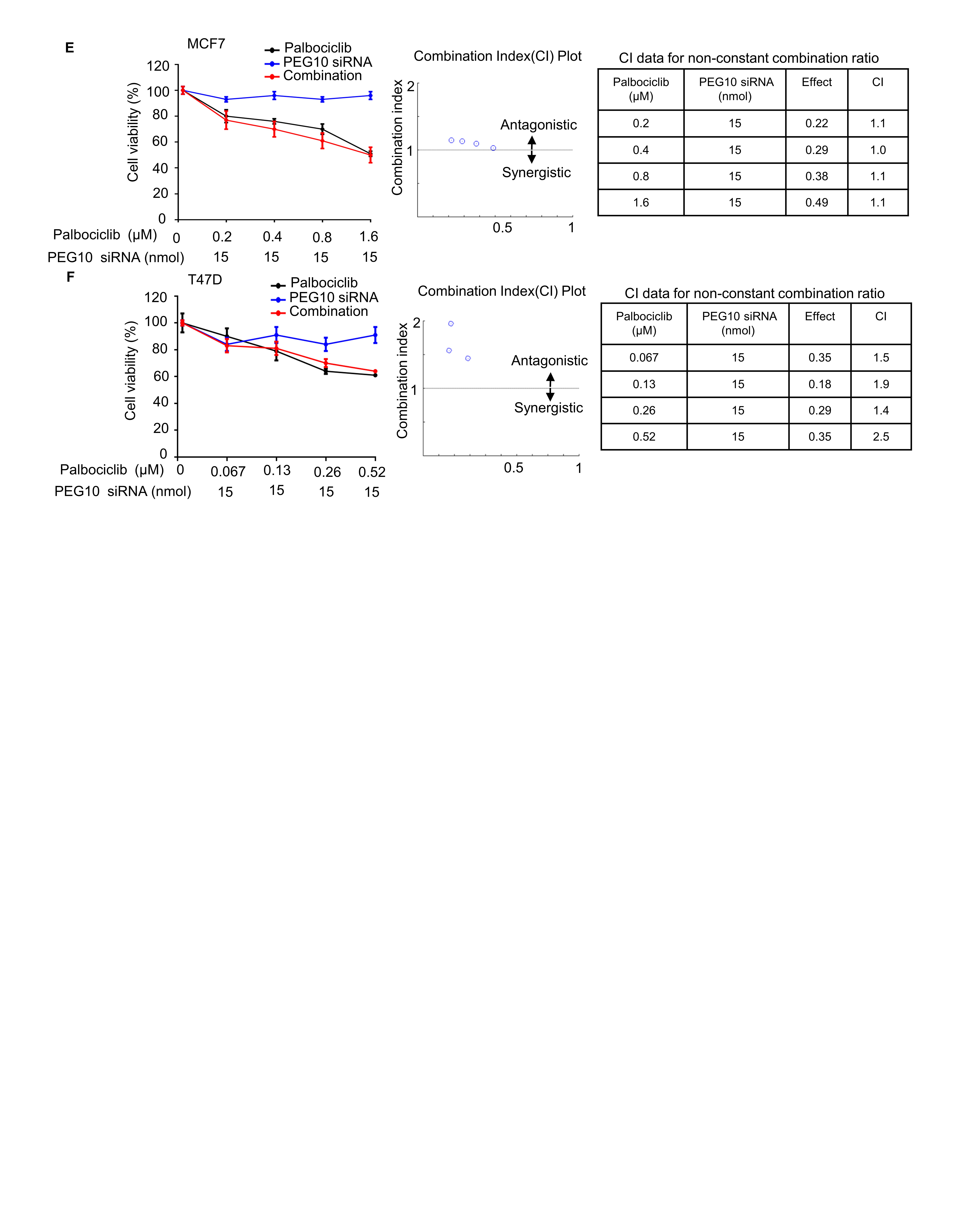


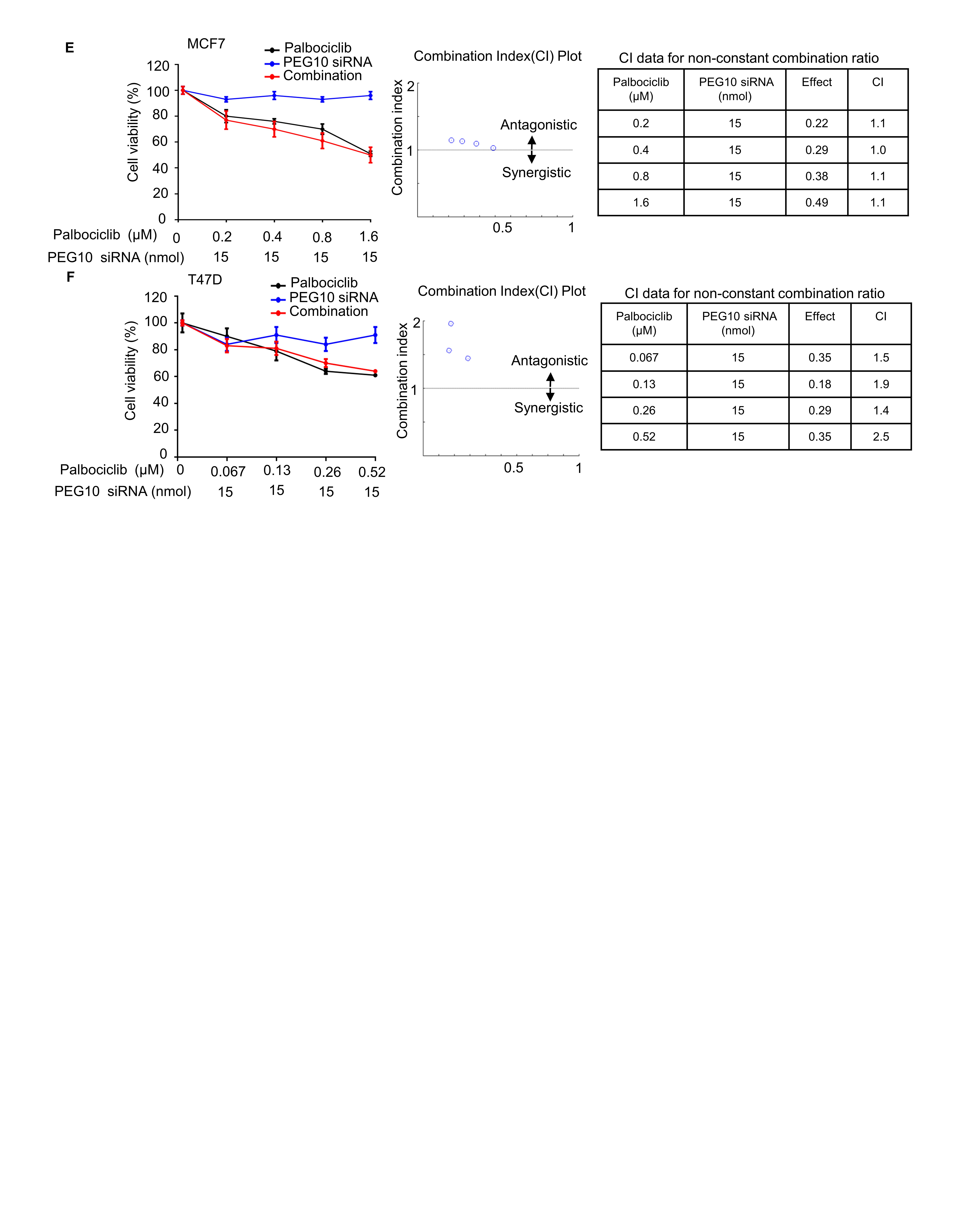


**Fig. S6.** (A) Cell viability (MTT) assay of MCF7-PR cells after treatment with PEG10 siRNA or abemaciclib and combination of various concentrations of abemaciclib and a fixed concentration of siRNA for 72 h. Three independently repeated experiments were performed with similar results. The CI values were calculated using the Chou–Talalay method. CI < 1 indicates synergism.

(B) Cell viability (MTT) assay of MCF7-PR cells after treatment with PEG10 siRNA or ribociclib and combination of various concentrations of ribociclib and a fixed concentration of siRNA for 72 h. Three independently repeated experiments were performed with similar results. The CI values were calculated using the Chou–Talalay method. CI < 1 indicates synergism.

(C) Cell viability (MTT) assay of T47D-PR cells after treatment with PEG10 siRNA or abemaciclib and combination of various concentrations of abemaciclib and a fixed concentration of siRNA for 72 h. Three independently repeated experiments were performed with similar results. The CI values were calculated using the Chou–Talalay method. CI < 1 indicates synergism.

(D) Cell viability (MTT) assay of T47D-PR cells after treatment with PEG10 siRNA or ribociclib and combination of various concentrations of ribociclib and a fixed concentration of siRNA for 72 h. Three independently repeated experiments were performed with similar results. The CI values were calculated using the Chou–Talalay method. CI < 1 indicates synergism.

(E-F) MTT assay of MCF7 and T47D cell lines after treatment with PEG10 siRNA or palbociclib and combination of various concentrations of palbociclib and a fixed concentration of siRNA for 72 h. Three independently repeated experiments were performed with similar results. The CI values were calculated using the Chou–Talalay method. CI > 1 and CI = 1 indicate synergism and additive effect, respectively.
