# Supplementary material for: Targeting PEG10 as a novel therapeutic approach to overcome CDK4/6 inhibitor resistance in breast cancer
Source: J Exp Clin Cancer Res. 2023 Nov 28;42:325. doi: 10.1186/s13046-023-02903-x (PMC10683152; doi:10.1186/s13046-023-02903-x)
Supplement: Supplementary file 7 — Additional file 7: Fig. S7. (A) Kaplan-Meier survival curves of RFS in HR+ breast cancer according to relative PEG10 mRNA expression from public mRNA microarray data sets of GSE 25066. Analysis of this data set was performed using the online platform Kaplan-Meier plotter (https://kmplot.com/analysis/). [file 13046_2023_2903_MOESM7_ESM.docx]

**Supplementary Figure S7**


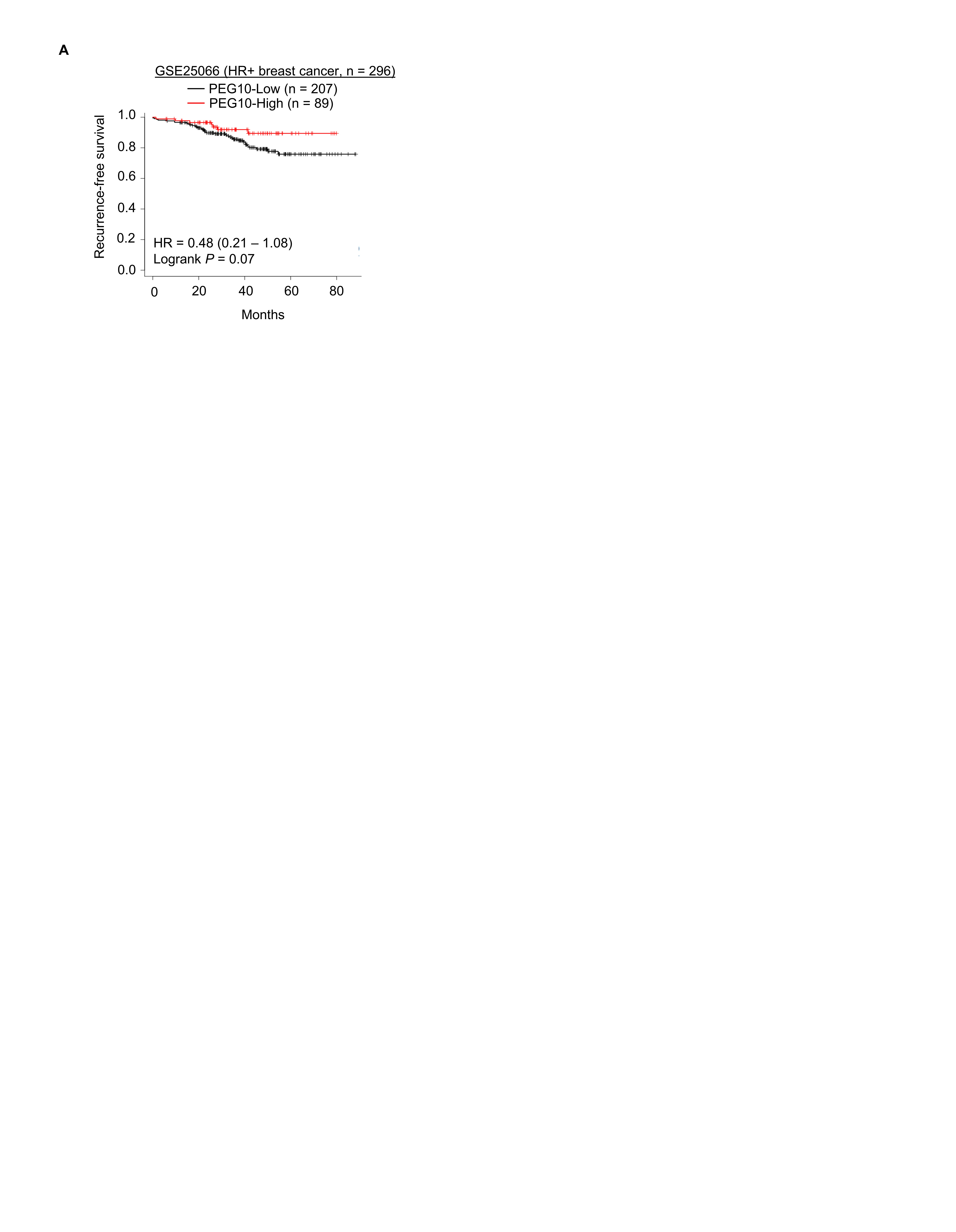


**Fig. S7. (A)** Kaplan-Meier survival curves of RFS in HR+ breast cancer according to relative PEG10 mRNA expression from public mRNA microarray data sets of GSE 25066. Analysis of this data set was performed using the online platform Kaplan-Meier plotter (<https://kmplot.com/analysis/>).
