# Supplementary material for: Targeting PEG10 as a novel therapeutic approach to overcome CDK4/6 inhibitor resistance in breast cancer
Source: J Exp Clin Cancer Res. 2023 Nov 28;42:325. doi: 10.1186/s13046-023-02903-x (PMC10683152; doi:10.1186/s13046-023-02903-x)
Supplement: Supplementary file 8 — Additional file 8: Table S1. List of primers used for qRT-PCR. Table S2. List of Primary and secondary antibodies used for western blot and immunohistochemistry. Table S3. list of 43 breast cancer cell lines in the GDSC database focusing on general subtypes and palbociclib IC50. Table S4. Clinical characteristics of patients whose tumors were used for PEG10 IHC. Table S5. List of the commonly upregulated genes in palbociclib-resistant cells compared with parental cell lines. Table S6. New drugs under clinical trials in the setting of CDK4/6 inhibitor resistance. [file 13046_2023_2903_MOESM8_ESM.docx]

**Supplementary Tables**

Table S1. List of primers used for qRT-PCR

| Primer name | Orientation | Sequence |
| --- | --- | --- |
| PEG10 | Sense | 5’-CTTCGAGAGCAAGTGGAACC-3’ |
|  | Anti-sense | 5’-CAGACACGGACACGATCAAC-3’ |
| ZEB1 | Sense | 5’-CAGCATCACCAGGCAGTCCC-3’ |
|  | Anti-sense | 5’-CAACAGCTTGCACCATGCCC-3’ |
| E-cadherin | Sense | 5’-GGTCGACAAAGGACAGCCTA-3’ |
|  | Anti-sense | 5’-GCGTGACTTTGGTGGAAAAC-3’ |
| N-cadherin | Sense | 5’-CAACCCCATCTCGGGTCAGC-3’ |
|  | Anti-sense | 5’-GGGCATTGGGATCGTCAGCA-3’ |
| Vimentin | Sense | 5’-CCCTCACCTGTGAAGTGGAT-3’ |
|  | Anti-sense | 5’-TCCAGCAGCTTCCTGTAGGT-3’ |
| SNAIL | Sense | 5’-CACTATGCCGCGCTCTTTC-3’ |
|  | Anti-sense | 5’-GGTCGTAGGGCTGCTGGAA-3’ |
| LAMC2 | Sense | 5’-GTCACTGGAGAACGCTGTGA-3’ |
|  | Anti-sense | 5’-GAGCTTTGCAGGAGACCCAT-3’ |
| Beta-actin | Sense | 5’-AGAGCTACGAGCTGCCTGAC-3’ |
|  | Anti-sense | 5’-AGCACTGTGTTGGCGTACAG-3’ |

Table S2. List of Primary and secondary antibodies used for western blot and immunohistochemistry.

| Antibody | Host species | Dilution | Company (catalog#) | Application |
| --- | --- | --- | --- | --- |
| PEG10 | Rabbit | 1:200 | 1442-1-AP, proteintech | IHC |
| PEG10 | Rabbit | 1:1000 | 77111, cell signaling | WB |
| ZEB1 | Rabbit | 1:1000 | ab124512, abcam | WB |
| E-cadherin | Rabbit | 1:1000 | 3195, cell signaling | WB |
| LAMC2 | mouse | 1:100 | sc-28330, santa cruz | WB |
| Caspase 3 | Rabbit | 1:1000 | 9662, cell signaling | WB |
| p21 | mouse | 1:100 | sc-6246, santa cruz | WB |
| SIAH1 | mouse | 1:100 | H00006477-MO2, abnova | WB |
| CDK2 | Rabbit | 1:1000 | 2546, cell signaling | WB |
| CyclinE | mouse | 1:1000 | 4129, cell signaling | WB |
| Cyclin D1 | Rabbit | 1:1000 | 55506, cell signaling | WB |
| Cyclin A | mouse | 1:100 | Sc-271682, santa cruz | WB |
| Ki67 | Rabbit | 1:200 | ab92742, abcam | IHC |
| CST/2368T / DYKDDDDTag antibody | Rabbit | 1:1000 | CST/2368, cell signaling | WB |
| β-actin | Rabbit | 1:1000 | sc-69879, santa cruz | WB |
| GAPDH | Rabbit | 1:1000 | 2118, cell signaling | WB |
| Anti-Rabbit HRP | Goat | 1:5000 | GTX213110-01, GeneTex | WB |
| Anti-Mouse HRP | Goat | 1:5000 | GTX213111-01, GeneTex | WB |
|  |  |  |  |  |

Abbreviations: IHC, immunohistochemistry; WB, western blot

Table S3. list of 43 breast cancer cell lines in the GDSC database focusing on general subtypes and palbociclib IC_50_.

| Cell line | Cosmic ID | Cancer type | General subtype | Palbociclib IC_50_ (µM) |
| --- | --- | --- | --- | --- |
| HCC1954 | 749709 | breast | HER2+ | 2.77 |
| HCC1143 | 749710 | breast | TNBC | 0.56 |
| HCC1187 | 749711 | breast | TNBC | 3.15 |
| HCC1395 | 749712 | breast | TNBC | 2.17 |
| HCC1937 | 749714 | breast | TNBC | 3.6 |
| HCC2157 | 749715 | breast | TNBC | 4.08 |
| HCC2218 | 749716 | breast | HER2+ | 4.61 |
| HCC38 | 749717 | breast | TNBC | 3.27 |
| T47D | 905945 | breast | HR+ | 0.19 |
| MCF7 | 905946 | breast | HR+ | 0.4 |
| BT-549 | 905951 | breast | TNBC | 4.81 |
| Hs-578-T | 905957 | breast | TNBC | -0.04 |
| MDA-MB-231 | 905960 | breast | TNBC | 0.74 |
| BT-20 | 906801 | breast | TNBC | 4.28 |
| COLO-824 | 906812 | breast | n/a | 4.17 |
| CAL-120 | 906826 | breast | TNBC | 1.73 |
| DU-4475 | 906844 | breast | TNBC | 3.29 |
| EVSA-T | 906862 | breast | ER-HER2-/+ | 1.88 |
| HCC1419 | 907045 | breast | HER2+ | 4.48 |
| HCC1569 | 907046 | breast | HER2+ | 4.16 |
| HCC1806 | 907047 | breast | TNBC | 1.44 |
| MDA-MB-361 | 908121 | breast | ER+PR+HER2+ | 1.85 |
| MDA-MB-453 | 908122 | breast | TNBC | 4.01 |
| MDA-MB-468 | 908123 | breast | TNBC | 3.67 |
| MRK-nu-1 | 908151 | breast | n/a | 2.71 |
| OCUB-M | 909256 | breast | n/a | 2.57 |
| ZR-75-30 | 909907 | breast | ER+PR-HER2+ | 5.48 |
| AU565 | 910704 | breast | HER2+ | 2.25 |
| CAL-85-1 | 910852 | breast | TNBC | 1.12 |
| CAL-51 | 910927 | breast | ER-HER2- | 0.34 |
| MFM-223 | 910948 | breast | TNBC | 1.19 |
| MDA-MB-415 | 924240 | breast | HR+ | 3.29 |
| MDA-MB-157 | 925338 | breast | TNBC | 2.92 |
| BT-474 | 946359 | breast | ER+PR+HER2+ | 3.94 |
| BT-483 | 949093 | breast | HR+ | 4.4 |
| MDA-MB-436 | 1240172 | breast | TNBC | 3.47 |
| EFM-192A | 1290798 | breast | HR+ | 2.45 |
| HCC1428 | 1290905 | breast | HR+ | 2.13 |
| HCC202 | 1290906 | breast | HER2+ | 4.95 |
| HDQ-P1 | 1290922 | breast | TNBC | 4.43 |
| JIMT-1 | 1298157 | breast | n/a | 2.61 |
| HCC1500 | 1303900 | breast | HR+ | 4.19 |
| MDA-MB-330 | 1330941 | breast | HR+ | 2.82 |

Abbreviations: ER, estrogen receptor; PR, progesterone receptor; HER2, human epidermal growth factor receptor 2; HR+, hormone receptor-positive; n/a, not applicable

Table S4: Clinical characteristics of patients whose tumors were used for PEG10 IHC

| Characteristics | Patients no. (total, n=15) | |
| --- | --- | --- |
| Age | |  |
| median (range), year | | 56 (42–85) |
| Subtype | |  |
| HR+HER2– | | 14 |
| HR+HER2+ | | 1 |
| Metastatic sites | |  |
| LN only | | 2 |
| Bone only | | 2 |
| Bone and skin | | 1 |
| Visceral (lung, liver, pleural, et al.) | | 10 |
| CDK4/6 inhibitor/endocrine therapy | |  |
| Palbociclib/ Letrozole or anastrozole | | 14 |
| Line of CDK4/6 inhibitor | |  |
| 1^st^ line | | 15 |
| Follow-up duration | |  |
| Median (range), month | | 10.0 (1.0–72.5) |
| Cause of stopping CDK4/6 inhibitor | |  |
| Ongoing | | 4 |
| Stopped | |  |
| Progression | | 10 |
| Adverse event | | 1 |

Abbreviations: HR+, hormone receptor-positive; HER2–, human epidermal growth factor receptor 2-negative; HER2+, human epidermal growth factor receptor 2-positive; LN, Lymph node

Table S5. List of the commonly upregulated genes in palbociclib-resistant cells compared with parental cell lines.

| Gene symbol | Fold change | |
| --- | --- | --- |
|  | MCF7-PR/ MCF7 | T47D-PR/ T47D |
| PEG10 | 3.24 | 7.75 |
| MNS1 | 2.27 | 3.84 |
| SDC2 | 3.29 | 3.58 |
| TRDJ4 | 3.81 | 2.90 |
| CGB8 | 3.81 | 2.90 |
| ZNF733P | 2.88 | 2.30 |
| MAPT-IT1 | 2.01 | 2.29 |
| SPDYE3 | 2.32 | 2.13 |
| C3orf80 | 2.32 | 2.13 |

A gene showing fold change ≥ 2 compared with that in parental cells (MCF7 and T47D) is considered as an upregulated gene.

Table S6. New drugs under clinical trials in the setting of CDK4/6 inhibitor resistance.

| Target | Phase | Drugs | Setting in breast cancer | References |
| --- | --- | --- | --- | --- |
| CDK7 | II | Samuraciclib ± endocrine | Prior CDK4/6 inhibitor | Coombes et al., 2022 ^1^ |
| CDK2/4/6 | I/2a | Ebvaciclib | Prior CDK4/6 inhibitor | Yap et al., 2022 ^2^ |
| FGFR | I | Erdafitinib + fulvestrant + palbociclib | Prior CDK4/6 inhibitor; FGFR-amplified | Mayer et.al., 2021 ^3^ |
| PI3K/AKT | III | Ipatasertib + fulvestrant | Progression on 1^st^ line CDK4/6 inhibitor and AI | Chia et al., 2023 ^4^ |

Abbreviation: AI, aromatase inhibitor

**SI References**

1. Coombes C, Howell SJ, Krebs MG, Lord S, Kenny LM, Bahl A*, et al.* Study of samuraciclib (CT7001), a first-in-class, oral, selective inhibitor of CDK7, in combination with fulvestrant in patients with advanced hormone receptor positive HER2 negative breast cancer (HR+BC). Abstract of the annual meeting of the American Association for Cancer Research. <https://doi.org/10.1158/1538-7445.SABCS21-GS3-10>. Accessed 2022 December 13.

2. Yap TA, Basu C, Goldman JW, Gordon M, Hamilton E, Kelly A*, et al.* A first-in-human phase 1/2a dose escalation/expansion study of the first-in-class CDK2/4/6 inhibitor PF-06873600 alone or with endocrine therapy in patients with breast or ovarian cancer. Abstract of the annual meeting of the American Association for Cancer Research. <https://doi.org/10.1158/1538-7445.SABCS21-P5-16-06>. Accessed 2022 December 18.

3. Mayer IA, Haley BB, Abramson VG, Brufsky A, Rexer B, Stringer-Reasor E*, et al.* Abstract PD1-03: A phase Ib trial of fulvestrant+ CDK4/6 inhibitor (CDK4/6i) palbociclib+ pan-FGFR tyrosine kinase inhibitor (TKI) erdafitinib in FGFR-amplified/ER+/HER2-negative metastatic breast cancer (MBC). Abstract of the annual meeting of the American Association for Cancer Research. Cancer Research 2021. <https://doi.org/10.1158/1538-7445.SABCS20-PD1-03>. Accessed 2023 January 6.

4. Chia SK, Cescon DW, Redfern AD, Rodin D, Simmons C, Ayoub J-P*, et al.* double-blind placebo controlled phase III trial of fulvestrant and ipatasertib for advanced HER-2 negative and estrogen receptor positive (ER+) breast cancer post line CDK4/6 and aromatase inhibitor therapy (FINER). Abstract of the annual meeting of the American Association for Cancer Research. Cancer Research 2022. <https://doi.org/10.1158/1538-7445.SABCS22-OT3-26-01>. Accessed at 2023 April 7.
